# Hydrogen Activation with Ru-PN^3^P Pincer Complexes for the Conversion of C_1_ Feedstocks

**DOI:** 10.1021/acs.inorgchem.3c04001

**Published:** 2024-02-08

**Authors:** Matthew D. Morton, Boon Ying Tay, Justin J.Q. Mah, Andrew J.P. White, James D. Nobbs, Martin van Meurs, George J.P. Britovsek

**Affiliations:** † Department of Chemistry, Imperial College London, Molecular Sciences Research Hub, White City Campus, 82 Wood Lane, London W12 0BZ, United Kingdom; ‡ Institute of Sustainability for Chemicals, Energy and Environment (ICSE2), Agency for Science, Technology and Research (A*STAR), 1 Pesek Road Jurong Island, Singapore 627833, Republic of Singapore

## Abstract

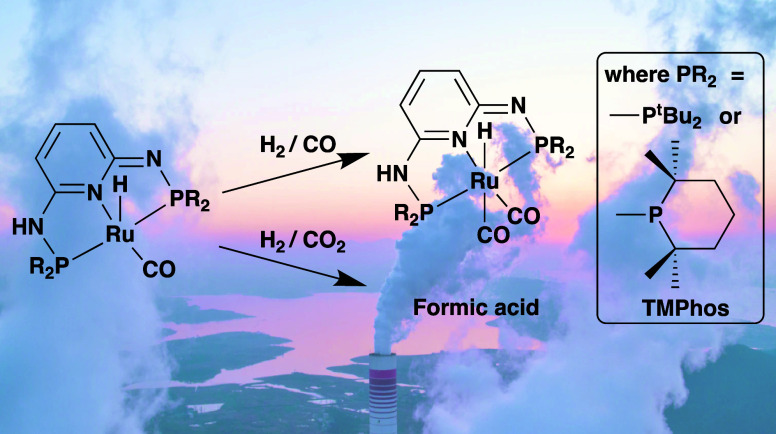

The hydrogenation of C_1_ feedstocks (CO and CO_2_) has been investigated using ruthenium complexes [RuHCl(CO)(PN^3^P)] as the catalyst. PN^3^P pincer ligands containing amines in the linker between the central pyridine donor and the phosphorus donors with bulky substituents (*tert*-butyl (**1**) or TMPhos (**2**)) are required to obtain mononuclear single-site catalysts that can be activated by the addition of KO^t^Bu to generate stable five-coordinate complexes [RuH(CO)(PN^3^P–H)], whereby the pincer ligand has been deprotonated. Activation of hydrogen takes place via heterolytic cleavage to generate [RuH_2_(CO)(PN^3^P)], but in the presence of CO, coordination of CO occurs preferentially to give [RuH(CO)_2_(PN^3^P–H)]. This complex can be protonated to give the cationic complex [RuH(CO)_2_(PN^3^P)]^+^, but it is unable to activate H_2_ heterolytically. In the case of the less coordinating CO_2_, both ruthenium complexes **1** and **2** are highly efficient as CO_2_ hydrogenation catalysts in the presence of a base (DBU), which in the case of the TMPhos ligand results in a TON of 30,000 for the formation of formate.

## Abstract 2

Hydrogenation of C_1_ feedstocks (CO and CO_2_) has been investigated using ruthenium complexes [RuHCl(CO)(PN^3^P)] as the catalyst precursor. PN^3^P ligands with bulky substituents are required to obtain mononuclear single-site hydrogenation catalysts. In the presence of CO, coordination of CO occurs preferentially to give [RuH(CO)_2_(PN^3^P−H)], which is unable to activate H_2_. In the case of the less coordinating CO_2_, the ruthenium complexes are highly efficient as CO_2_ hydrogenation catalysts.

## Introduction

The efficient production of green hydrogen and its conversion to renewable liquid energy carriers such as formic acid or methanol, so-called power-to-liquids (P2L) processes, are gaining increased interest due to the urgent need for sustainable fuels.^1−3^ Green hydrogen refers to H_2_ obtained from a renewable source, for example, through water electrolysis using renewable electricity or from the gasification of biomass. Hydrogenations of C_1_ feedstocks such as CO or CO_2_ are important in this context as this can give access to a range of green chemicals, including HCO_2_H, MeOH, and hydrocarbons.^4−8^ The hydrogenation of CO is currently applied in several large-scale chemical processes such as the Fischer–Tropsch process and the production of MeOH (110 Mt per annum),^9^ all based on heterogeneous catalysis. Likewise, the industrial hydrogenation of CO_2_ to CH_4_^10^ or MeOH^11^ also utilizes heterogeneous catalysis. Although at a much smaller scale of deployment as compared to that of CO, CO_2_ is becoming an increasingly important feedstock. The synthesis of MeOH appears to be the most attractive, exemplified by the 5,000 tons per annum plant operated by CRI in Iceland and other demonstration plants.^11,12^ With advances in the efficiencies of electrolyzers and hydrogenation technologies, CO_2_ hydrogenation to green methanol or dimethyl ether (DME) is likely to become increasingly important in the near future. Longer term, this could result in a MeOH-based economy as originally envisaged by Asinger in 1986,^13^ and later also by Olah et al.^14^

Efficient homogeneous systems capable of hydrogenation reactions of these C_1_ feedstocks have long remained elusive, despite the heterogeneous systems being well established, in some cases for more than a century. However, recently there have been some exciting discoveries of molecular catalysts that can reduce CO to MeOH via formamide or formate intermediates.^15−17^ These discoveries have occurred in tandem with significant advances in homogeneous hydrogenation of CO_2_ to MeOH in the past decade.^18^

**Scheme 1:**
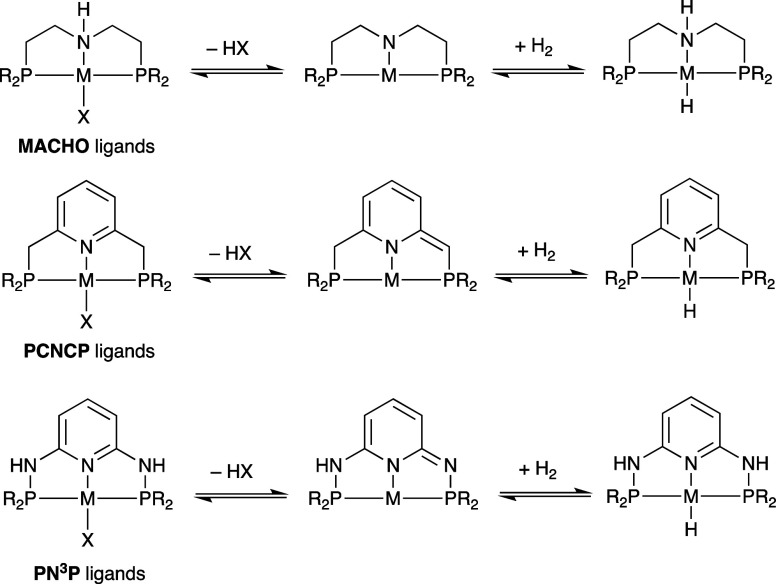
Reversible Deprotonation and H_2_ Activation through Metal Ligand Cooperativity in **MACHO**, **PCNCP**, and **PN**^**3**^**P** Pincer Complexes

The catalytic hydrogenation of CO and CO_2_ feedstocks both require heterolytic cleavage of H_2_ to generate hydride and proton donors. Pincer ligands of the types **PCNCP** and **PN**^**3**^**P** such as those shown in Scheme 1 and similar PNN systems,^19^ as well as the related **MACHO** ligand types,^20−24^ have featured prominently in this area as they can be reversibly protonated and deprotonated.^25−30^ This has made these ligands particularly attractive for hydrogenations using H_2_ activation reactions via metal–ligand cooperative (MLC) behavior,^31−34^ although recent discoveries suggests that this MLC behavior may not be essential for all catalyst systems.^23^ Here, we present our studies on the synthesis of a series of ruthenium(II) complexes featuring PN^3^P-R ligands (where R = ^t^Bu and TMPhos^35^) and their reactivity toward H_2_ and CO, as well as their application in the hydrogenation of CO and CO_2_.

## Results and Discussion

### Synthesis and reactivity of PN^3^P Ru complexes

**Scheme 2:**
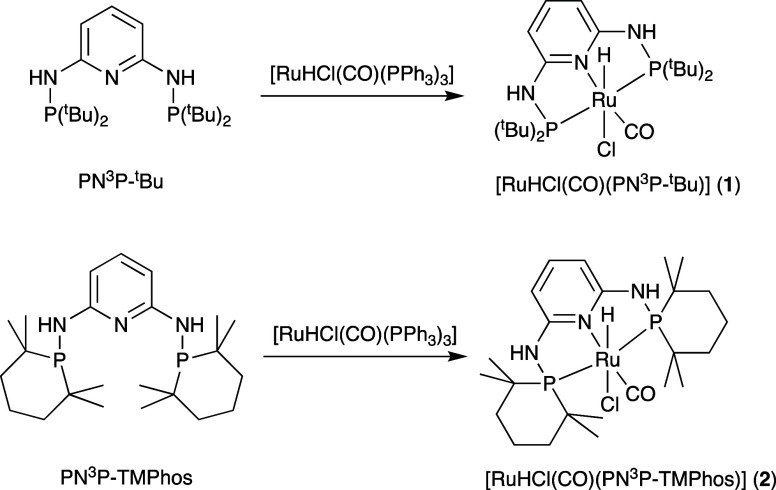
Synthesis of Ru-PN^3^P Complexes **1** and **2**

[RuHCl(CO)(PN^3^P-^t^Bu)] (**1**) was prepared by mixing a PN^3^P-^t^Bu ligand with [RuHCl(CO)(PPh_3_)_3_] in THF according to the procedure described by Huang et al. and shown in Scheme 2.^36^ The ^1^H NMR spectrum (*d*_4_-MeOH) shows a distinctive hydride signal at −24.1 ppm (triplet, ^2^*J*_HP_ = 18 Hz) and a doublet in the ^31^P NMR spectrum at 134 ppm (^2^*J*_PH_ = 18 Hz). In *d*_6_-benzene or CDCl_3_ instead of *d*_4_-MeOH, the NH protons are observed as a broad singlet. The IR spectrum shows a CO stretch at 1936 cm^–1^, and the Ru–H stretch is seen at 2113 cm^–1^. All data is shown in the Supporting Information and is consistent with that reported by Huang and co-workers.^36^

An alternative bulky phosphine 2,2,6,6-tetramethylphosphinane (TMPhos), recently developed by some of us,^35^ was also investigated as it provides a similar but subtly different stereoelectronic environment compared to P^t^Bu_2_ groups. The new ligand PN^3^P-TMPhos was obtained in 47% yield from 2,6-diaminopyridine and chloro-TMPhos (1-chloro-2,2,6,6-tetramethylphosphinane). The reaction with PN^3^P-TMPhos proceeds in a similar fashion as above resulting in [RuHCl(CO)(PN^3^P-TMPhos)] (**2**) (Scheme 2). Interestingly, the hydride signal appears much further downfield in this case at −14.4 ppm (^2^*J*_HP_ = 20 Hz, *d*_3_-MeCN), together with a significantly lower Ru–H stretching frequency at 2070 cm^–1^. The ^31^P NMR signal at 124 ppm and the carbonyl stretch at 1932 cm^–1^ are comparable to those of [RuHCl(CO)(PN^3^P-^t^Bu)] (**1**).

Similar reactions using the related PN^3^P-^*i*^Pr and -Ph ligands were also attempted but were largely unsuccessful. The reaction of PN^3^P-^*i*^Pr with [RuHCl(CO)(PPh_3_)_3_] in refluxing THF led to a mixture of the desired complex [RuHCl(CO)(PN^3^P-^*i*^Pr)] and presumably the cationic complex [RuH(CO)(PPh_3_)(PN^3^P-^*i*^Pr)]Cl, as well as other unidentified byproducts (see Figures S23 and S24). The reaction between PN^3^P-Ph and [RuHCl(CO)(PPh_3_)_3_] gave the cationic complex [RuH(CO)(PPh_3_)(PN^3^P-Ph)]Cl with some unidentified species (Figures S26 and S27). Similar issues in the synthesis of Ru complexes with the PN^3^P-Ph ligand have been reported, and a dinuclear Ru complex with a bridging PN^3^P ligand was isolated.^26^ Kirchner and co-workers synthesized the [(PN^3^P)RuCl_2_] complexes from [RuCl_2_(PPh_3_)_3_], which avoided the CO ligand.^37^

**Scheme 3:**
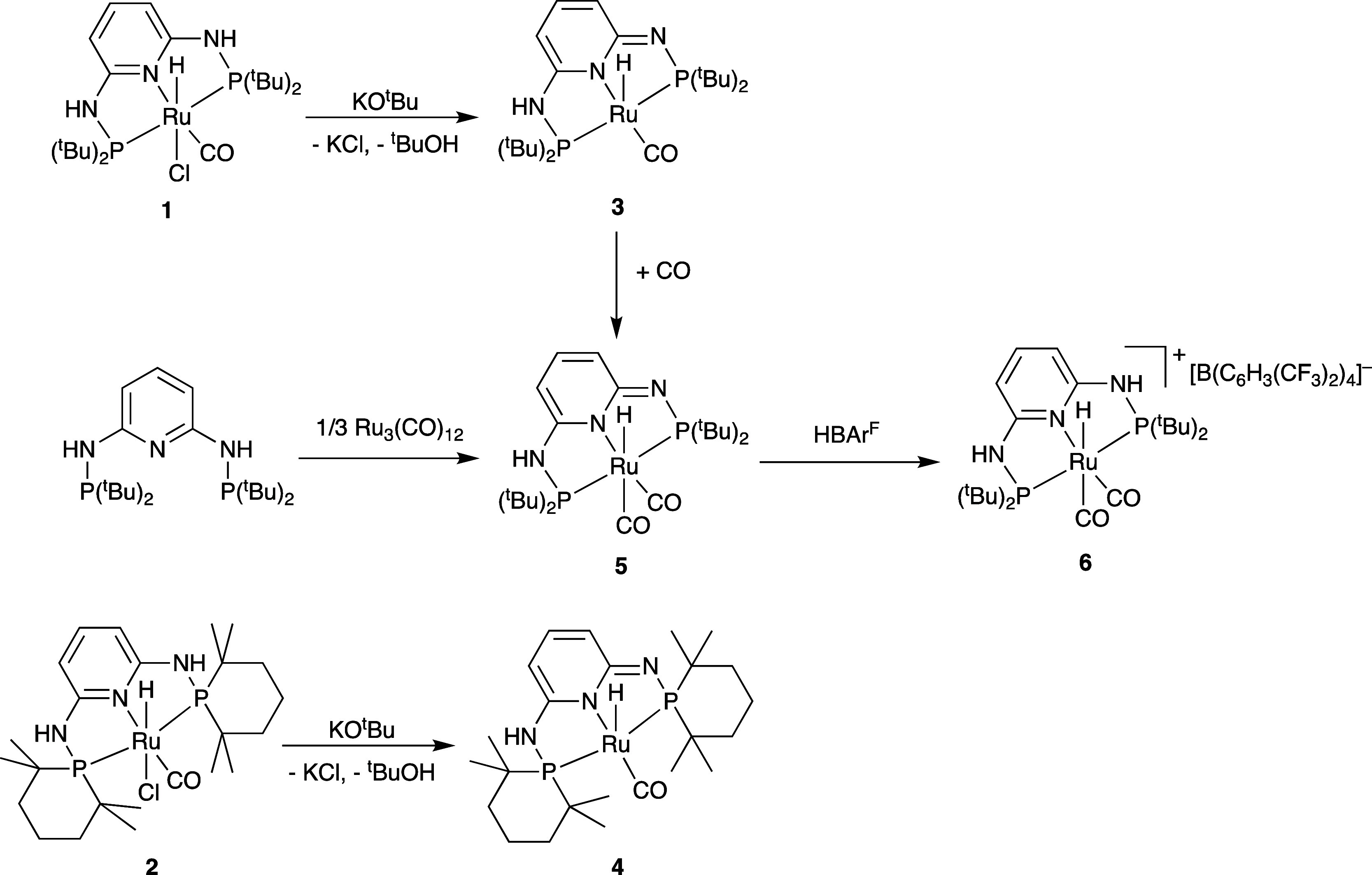
Reaction of Complexes **1** and **2** with KO^t^Bu and Subsequent Reactivity

Treatment of [RuHCl(CO)(PN^3^P-^t^Bu)] (**1**) with KO^t^Bu results in the formation of the five-coordinate complex [RuH(CO)(PN^3^P-^t^Bu–H)] (**3**) (Scheme 3). The ^1^H NMR spectrum of [RuH(CO)(PN^3^P-^t^Bu–H)] in *d*_6_-benzene shows a loss of symmetry in the aromatic region, and the hydride signal is shifted upfield to −25.9 ppm (^2^*J*_HP_ = 16 Hz) (Figure S5). The ^31^P{^1^H} NMR spectrum shows two doublets at 128.2 and 130.7 ppm (^2^*J*_PP_ = 220 Hz). The IR spectrum shows a single CO stretch at 1885 cm^–1^, which is 50 cm^–1^ lower than that for [RuHCl(CO)(PN^3^P-^t^Bu)] (**1**), suggesting greater back-bonding to CO. Similarly, [RuH(CO)(PN^3^P-TMPhos–H)] (**4**) can be generated from [RuHCl(CO)(PN^3^P-TMPhos)] (**2**) and 1 equiv of KO^t^Bu. The protons in the dearomatized pyridine ring see an upfield shift from 7.16 and 6.72 ppm to 6.83 and 5.77 ppm, respectively, while the hydride signal shifts slightly upfield from −14.4 to −14.5 ppm (^2^*J*_HP_ = 19 Hz). The ^31^P{^1^H} NMR spectrum shows a broad singlet at 122.1 ppm in contrast to the two doublets observed for the ^t^Bu complex **3**, which suggests fluxional behavior in CD_3_CN, similar to that observed for the CO-coordinated complex **5** (*vide infra*). We found that complex **4** is thermally unstable and gradually decomposes in solution.

The reaction of [RuH(CO)(PN^3^P-^t^Bu–H)] (**3**) with CO (1 bar) at room temperature (RT) in C_6_D_6_ resulted immediately in a color change from red to pink/orange and the formation of a dicarbonyl complex [RuH(CO)_2_(PN^3^P-^t^Bu–H)] (**5**) (Scheme 3). Complex **5** is also obtained from the reaction between PN^3^P-^t^Bu and [Ru_3_(CO)_12_] in toluene at 110 °C. In contrast, the reaction of PN^3^P-^*i*^Pr with [Ru_3_(CO)_12_] in refluxing toluene resulted in complicated mixtures of products (see Figure S25). The ^1^H NMR spectrum in C_6_D_6_ of [RuH(CO)_2_(PN^3^P-^t^Bu–H)] (**5**) is similar to the spectrum of [RuH(CO)(PN^3^P-^t^Bu–H)] (**3**) (see Figure S32), but the signals for the aromatic protons in 3 and 5 position and the *tert*-butyl groups are all broader. The hydride appears as a triplet at −6.1 ppm (^2^*J*_HP_ = 19 Hz), significantly downfield from −25.9 ppm in starting monocarbonyl complex **3**, probably due to the *trans* effect of the second carbonyl ligand. The ^31^P{^1^H} NMR spectrum (C_6_D_6_) shows two doublets at 135.2 and 140.9 ppm (^2^*J*_PP_ = 190 Hz), which are also broadened. There are two ν_(CO)_ absorptions at 1989 and 1940 cm^–1^ in the IR spectrum.

**Figure 1:**
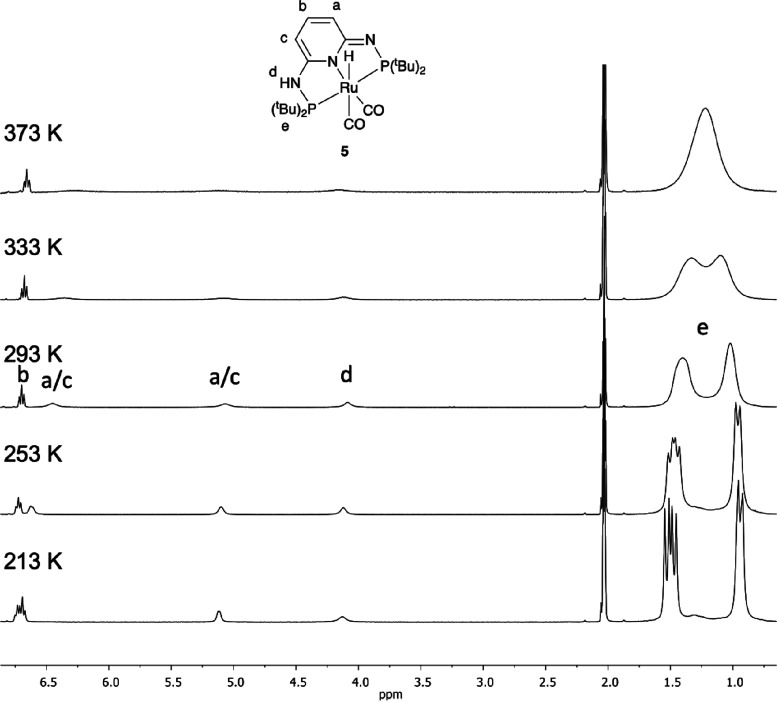
Variable temperature ^**1**^H NMR spectra of [RuH(CO)_2_(PN^**3**^P-^*t*^Bu–H)] (**5**) in *d*_8_-toluene (hydride signal has been omitted).

The dicarbonyl complex [RuH(CO)_2_(PN^3^P-^t^Bu-H)] (**5**) shows fluxional behavior in solution at RT, which was investigated by variable temperature ^1^H NMR spectroscopy in *d*_8_-toluene (see Figure 1). At 213 K, the broad peaks are better resolved although not completely sharp. Increasing the temperature results in further broadening and coalescence of the *tert*-butyl groups at 373 K. The ^31^P NMR spectra show two sharp doublets at low temperatures and two broad doublets at higher temperatures, but no coalescence is observed up to 373 K.

**Scheme 4:**
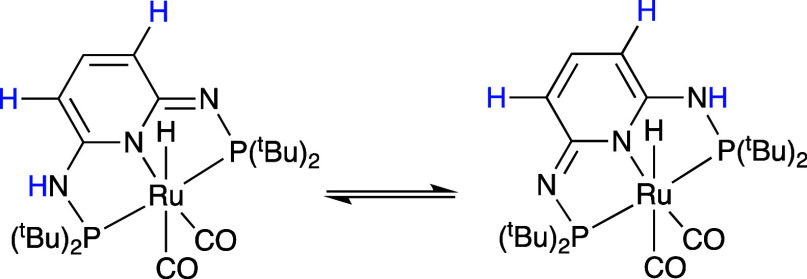
Equilibrium between the Two [RuH(CO)_2_(PN^**3**^P-^*t*^Bu–H)] (**5**) Isomers (Protons in Blue Show Exchange Behavior)

At all measured temperatures, the ^1^H NMR spectra show the pyridine proton in the 4 position as a triplet at 6.7 ppm and the hydride as a triplet at −6 ppm, and both are sharp signals. The fluxionality is proposed to involve proton transfer between the imine and amine moieties of the ligand, interconverting between the two enantiomeric forms of the complex, as shown in Scheme 4. Noteworthy, there was no fluxional behavior reported for the related complex [RuH(CO)(PPh_3_)(PN^3^P-Ph–H)].^26^

No evidence for an intermediate was observed, for example, a Ru(0) complex [Ru(CO)_2_(PN^3^P-^*t*^Bu)], which would be structurally similar to the [Ru(CO)_2_(PONOP-^*t*^Bu)] complex reported by Milstein et al.^38^ The ^1^H and ^31^P NMR spectra of [RuH(CO)_2_(PN^3^P-^*t*^Bu–H)] (**5**) were also measured in *d*_4_-methanol, where complete coalescence for the ^t^Bu signals is observed at RT. This indicates that the fluxional process most likely involves proton exchange with methanol facilitating the proton transfer from one side of the ligand to the other. The free energy of activation (Δ*G*^‡^) for the exchange process in *d*_8_-toluene was determined by NMR as 73 kJmol^–1^ (see the Supporting Information for details).

**Figure 2:**
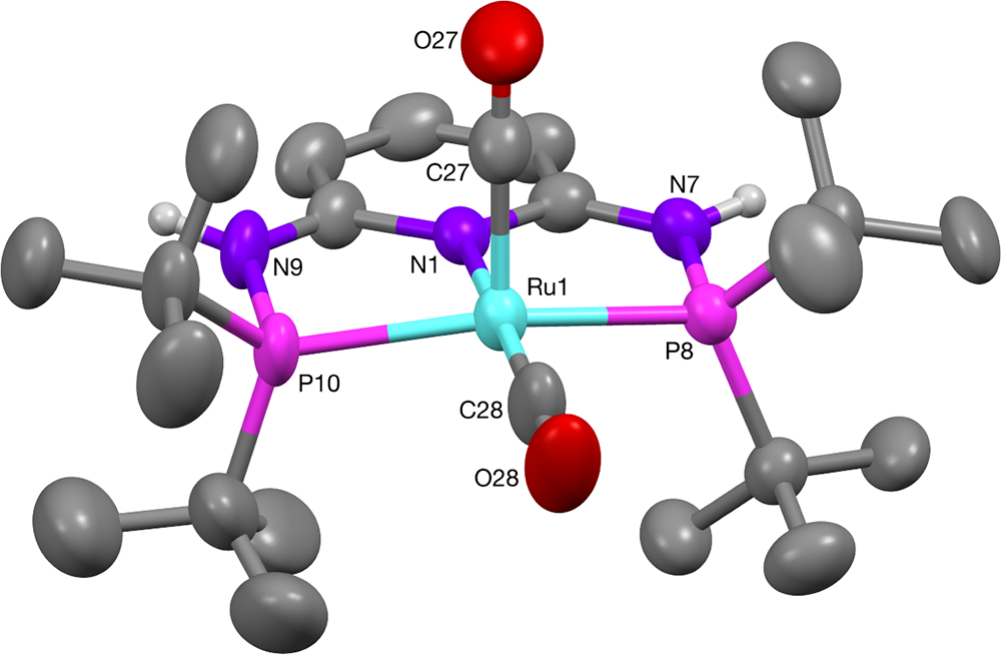
Molecular structure of the cationic complex [RuH(CO)_2_(PN^**3**^P-^*t*^Bu)]^+^ (**6**). The exact position of the hydride ligand could not be determined (Supporting Information). The anion [B(C_6_H_3_(CF_3_)_2_)_4_]^−^ has been omitted for clarity. Selected bond lengths (Å) and angles (deg): Ru(1)–C(28) 1.878(8), Ru(1)–C(27) 1.976(8), Ru(1)–N(1) 2.115(4), Ru(1)–P(8) 2.345(3), Ru(1)–P(10) 2.357(3); C(28)–Ru(1)–C(27) 95.6(4), P(8)–Ru(1)–P(10) 156.02(15), and C(28)–Ru(1)–N(1) 175.1(4).

Protonation of [RuH(CO)_2_(PN^3^P-^*t*^Bu–H)] (**5**) with a strong acid [H(OEt_2_)_2_][B(C_6_H_3_(CF_3_)_2_)_4_] (HBAr^F^) in THF results in the cationic complex [RuH(CO)_2_(PN^3^P-^*t*^Bu)]^+^ (**6**) in good yield (78%). The ^1^H NMR spectrum (C_6_D_5_Cl) is consistent with the rearomatization of the complex, resulting in a symmetric complex with only one signal for the protons in 3 and 5 position and a singlet in the ^31^P{^1^H} NMR spectrum at 142.7 ppm. No fluxional behavior was seen for this complex. The CO stretches in the IR spectrum are at 2017 and 1986 cm^–1^, which are 40 cm^–1^ higher than for the neutral complex [RuH(CO)_2_(PN^3^P-^*t*^Bu–H)] (**5**). The cationic complex **6** was further characterized by ^13^C NMR spectroscopy, mass spectrometry, elemental analysis, and SC-XRD (see the Supporting Information and Figure 2). The octahedral complex shows different Ru–C bond lengths, whereby the axial Ru–C bond *trans* to the hydride ligand is significantly longer at 1.976(8) Å, compared to the equatorial Ru–C bond at 1.878(8) Å. Related cationic complexes [RuH(CO)_2_(PNP-R)]^+^ with a different PNP-pincer-type ligand that features a central NH donor have been reported.^20,39^ In all cases, the Ru–C bond length of the axial CO ligand is significantly longer than that of the equatorial CO ligand by approximately 0.1 Å due to the *trans* hydride ligand.

### Hydrogen and CO Activation

**Scheme 5:**
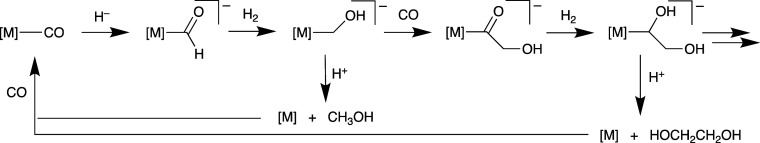
Proposed Reaction Sequence for the Hydrogenation of CO to MeOH and Higher Alcohols

The hydrogenation of CO with homogeneous transition metal-based catalysts to generate MeOH and further homologation products such as ethylene glycol is generally believed to proceed via the reaction sequence shown in Scheme 5. Formyl and hydroxymethyl complexes are generally invoked as intermediates in these reactions.^40−42^ Transition metal hydrides have been used by Bercaw et al. in homogeneous syngas conversion in combination with metal carbonyl complexes based on group 7 metals Re^43^ and Mn.^44^

**Scheme 6:**
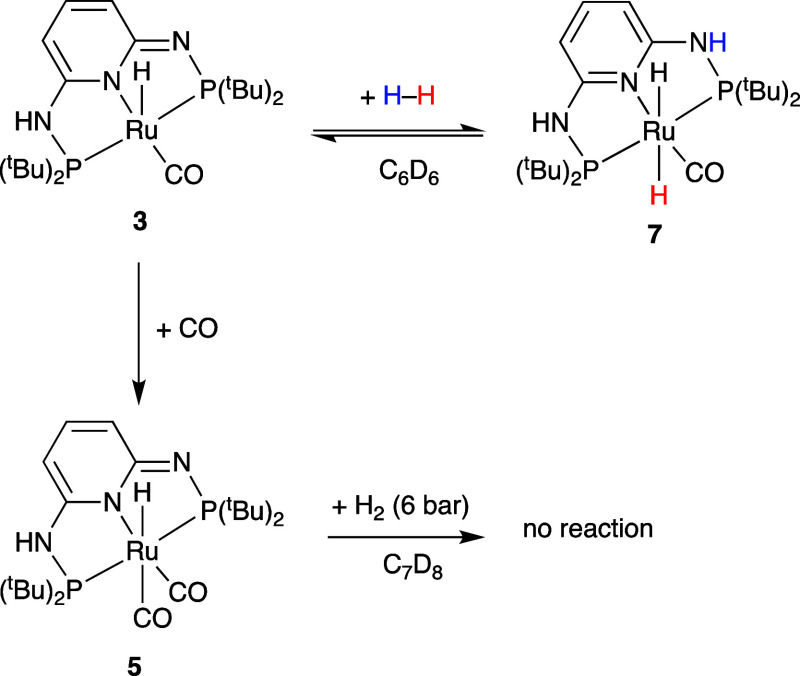
Reactivity of Complex **3** toward H_2_ in the Absence and Presence of CO

The reactivity of [RuH(CO)(PN^3^P-^t^Bu–H)] (**3**) toward H_2_ was explored in a series of stoichiometric reactions. The reaction between **3** and H_2_ (5 bar) was monitored by ^1^H and ^31^P NMR spectroscopy in C_6_D_6_. Immediately after the addition of H_2_, a new triplet is observed in the ^1^H NMR spectrum at −5.3 ppm (^2^*J*_HP_ = 19 Hz) and a new set of aromatic signals appears as a triplet and a doublet in a 1:2 ratio. A singlet is observed in the ^31^P{^1^H} NMR spectrum at 156.2 ppm. However, this reaction is slow, as noted by Huang et al.,^45^ and only approximately 1% conversion was observed after 1 h, but after 4 days at RT under H_2_, this increased to 10% (see Figures S19 and S20). Based on these observations, we assign the new signals to the symmetric *trans*-dihydride complex [Ru(H)_2_(CO)(PN^3^P-^t^Bu)] (**7**). Heating to 60 °C for 30 min resulted in decomposition of [RuH(CO)(PN^3^P-^t^Bu–H)] (**3**) rather than further reaction with H_2_ to generate more **7**. Repeating the experiment above with D_2_ instead of H_2_ resulted in a gradual decrease in the intensity of the hydride and the NH signals relative to the aromatic proton signals. This indicates that the H_2_ activation is reversible and there is an equilibrium between the two complexes in solution under H_2_, as indicated in Scheme 6. A direct synthesis of complex [Ru(H)_2_(CO)(PN^3^P-^t^Bu)] (**7**) was attempted by reacting complex [RuHCl(CO)(PN^3^P-^t^Bu)] (**1**) with NaBEt_3_H in toluene, as reported for the related [Ru(H)_2_(CO)(PONOP-^t^Bu)] complex,^38^ but the major product observed in this case was the five-coordinate complex [RuH(CO)(PN^3^P-^t^Bu–H)] (**3**). It is possible that complex **7** was formed as an intermediate but reverts immediately back to complex **3** in the absence of excess H_2_. The reaction of the dicarbonyl complex [RuH(CO)_2_(PN^3^P-^t^Bu–H)] (**5**) with H_2_ (6 bar, in *d*_8_-toluene) was unsuccessful, and no reaction was observed during 48 h at RT or after 30 min at 60 °C.

**Scheme 7:**
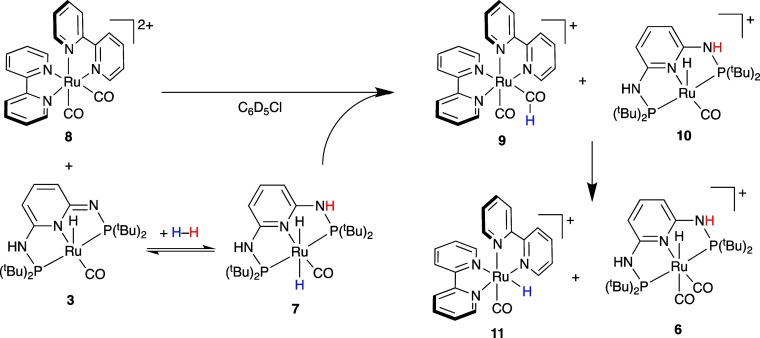
Hydride Transfer to Generate Metal Formyl Intermediates and Subsequent Decarbonylation

We subsequently explored the possibility of using a dual catalyst system, where one complex activates H_2_ and the other activates CO. The first system combines the ability of [RuH(CO)(PN^3^P-^t^Bu–H)] (**3**) to activate H_2_ and subsequently engage in intermolecular hydride transfer to other carbonyl complexes. Initially, we chose the Ru-bipy complex [Ru(bipy)_2_(CO)_2_](B(C_6_F_5_)_4_)_2_ (**8**) as a previous work by Tanaka and co-workers has shown that the formation of the formyl complex [Ru(bipy)_2_(CO)(CHO)]^+^ can be achieved by stoichiometric borohydride reduction.^46^ In an attempt to obtain a similar reduction using H_2_ instead of NaBH_4_, we reacted [Ru(bipy)_2_(CO)_2_](B(C_6_F_5_)_4_)_2_ (**8**) with [RuH(CO)(PN^3^P-^t^Bu–H)] (**3**) in the presence of H_2_ (4 bar) in C_6_D_5_Cl at RT according to Scheme 7.

The reaction of [Ru(bipy)_2_(CO)_2_](B(C_6_F_5_)_4_)_2_ (**8**) and [RuH(CO)(PN^3^P-^t^Bu–H)] (**3**) with H_2_ leads to an instant conversion to the formyl complex [Ru(bipy)_2_(CO)(CHO)]^+^ (**9**) as indicated by the characteristic singlet at 13.9 ppm^47^ together with the cationic Ru complex **10** (see Figure S33). This signal disappears over the course of several hours due to decarbonylation to form [Ru(bipy)_2_(CO)(H)]^+^ (**11**) evidenced by the emergence of the Ru-hydride signal at −11.4 ppm^48^ and [RuH(CO)_2_(PN^3^P-^t^Bu)]^+^ (**6**) at −6.7 ppm. The thermal instability of the formyl complex [Ru(bipy)_2_(CO)(CHO)]^+^ (**9**) has been noted previously.^47^ Another experiment was performed by reacting [Ru(bipy)_2_(CO)_2_](B(C_6_F_5_)_4_)_2_ (**8**) and [RuH(CO)(PN^3^P-^t^Bu–H)] (**3**) with syngas (CO/H_2_ = 3/1), which formed [RuH(CO)_2_(PN^3^P-^t^Bu–H)] (**5**) instantaneously, precluding any further heterolytic cleavage of H_2_.

**Scheme 8:**
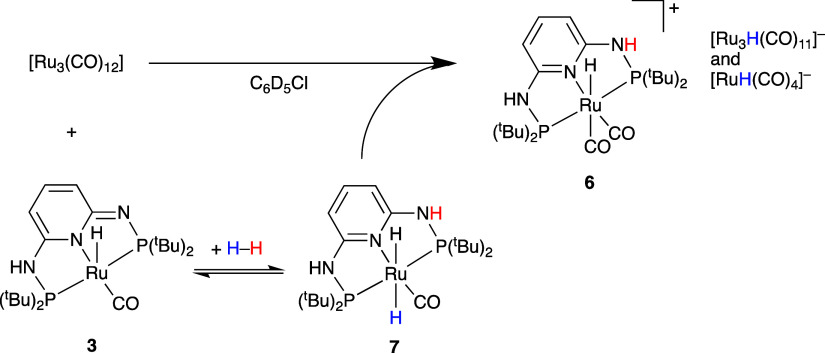
Hydride Transfer to [Ru_3_(CO)_12_]

Inspired by Dombek’s early observations on the formation of MeOH and ethylene glycol from [Ru_3_(CO)_12_] and syngas,^49^ albeit under forcing conditions, we attempted the hydrogenation of [Ru_3_(CO)_12_] in the presence of [RuH(CO)(PN^3^P-^t^Bu–H)] (**3**) at RT (Scheme 8). Upon introduction of H_2_ (1 bar), fast H_2_ activation to give [RuH_2_(CO)(PN^3^P-^t^Bu)] (**7**) was followed by a slow hydride and CO exchange with [Ru_3_(CO)_12_] to generate the known anionic complexes [Ru_3_H(CO)_11_]^−^ and [RuH(CO)_4_]^−^.^50,51^ It is possible that this exchange also involves metal formyl species as intermediates. Before H_2_ addition, some CO exchange was observed to form small amounts of [RuH(CO)_2_(PN^3^P-^t^Bu–H)] (**5**) (see Figure S34).

From these stoichiometric reactivity studies, it can be concluded that pincer complexes such as [RuH(CO)(PN^3^P-^t^Bu–H)] (**3**) are able to activate H_2_ to give [Ru(H)_2_(CO)(PN^3^P-^t^Bu)] (**7**), but no hydride transfer to the coordinated carbonyl ligand is observed. The activated complex **7** is able to transfer hydride equivalents to other metal carbonyl complexes to generate metal formyl intermediates, but these tend to be unstable at higher temperatures. More importantly, when using syngas, H_2_ activation becomes inhibited due to the preferential coordination of CO. Higher partial pressures of H_2_ may be needed to overcome this and displace coordinated CO. The hydrogenation of CO_2_ however should proceed as long as no CO is present as one of the reaction products.

### Hydrogenation of CO_2_ to Formate

Huang and co-workers have shown previously that Ru complex **1** is an efficient catalyst for H_2_ generation from the decomposition of HCO_2_H (TON > 1 million).^45^ Since equimolar CO_2_ is also generated, the reverse reaction, i.e., hydrogenation of CO_2_, would be needed for HCO_2_H to be feasible as a renewable H_2_/energy carrier. However, direct conversion of CO_2_ to formic acid is endergonic and therefore a base or high pressures are usually employed to drive the forward reaction.^6,7,52^ Pidko and co-workers have demonstrated that related Ru-PNP-pincer catalysts are highly efficient in the base-assisted hydrogenation of CO_2_ to formates with reported mol DBU-formate/(mol cat x h) (TOFs) in excess of 1 million h^–1^ at exceptionally low Ru loadings (<0.2 μmol).^25^

We have investigated complexes **1** and **2** as catalysts for the CO_2_ hydrogenation reaction, and the results are shown in Table 1. In a typical experiment, the precatalyst, dissolved in the reaction solvent dimethylformamide (DMF), was introduced into a batch reactor together with the base 1,8-diazabicyclo[5.4.0]undec-7-ene (DBU). After heating to the reaction temperature (90 °C), a mixture of CO_2_/H_2_ (7.5/7.5 bar) was introduced into the reactor and maintained at this pressure throughout the reaction. In the absence of any catalyst (entry 1), negligible gas consumption was observed, although a small amount of white solid was formed, likely to be DBU-bicarbonate [(DBU)H]^+^[HCO_3_]^−^ resulting from trace amounts of moisture in the system.^53^ Gratifyingly, both catalysts **1** and **2** were highly efficient in the DBU-assisted hydrogenation of CO_2_ to formate. Quantitative conversion of DBU was observed within 1 h at catalyst loadings of >5 μmol (entries 2–5). At a catalyst loading of 14.2 μmol, catalyst **1** and **2** gave TONs of 4400 and 4700, respectively, reflecting >93% conversion of DBU. Even when the catalyst loading was lowered to 5.7 μmol, full conversion of DBU was observed for both complexes **1** and **2** equating to TONs of 11,800 (entries 4 and 5).

**Table 1:**
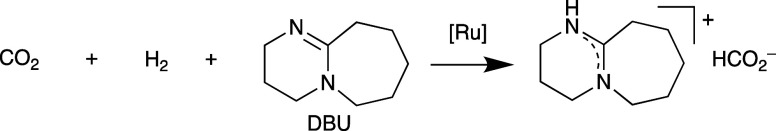
Base-Assisted Hydrogenation of CO_2_ to Formate Using Complexes **1** and **2**^b^

entry	catalyst	[Ru] μmol	conversion^a^ (%)	TON^b^
1	blank	0	0	0
2	**1**	14.2	93	4400
3	**2**	14.2	>99	4700
4	**1**	5.7	>99	11,800
5	**2**	5.7	>99	11,800
6	**1**	2.8	10	2300
7	**2**	2.8	100	23,600
8	**1**	1.4	0.3	100
9	**2**	1.4	64	30,300

a conversion = DBU-formate/DBU × 100.

b TON = mol DBU-formate/mol cat.

c Conditions: CO_2_/H_2_ (1:1) = 15 bar, temp. = 90 °C, reaction time = 1 h, DBU = 10 mL (66.9 mmol), solvent = DMF (35 mL).

Since the reaction is limited by the amount of DBU, we lowered the catalyst loading further in order to differentiate between the performances of the two catalysts. At a lower catalyst loading of 2.8 μmol, using complex **1**, conversion of DBU decreased to 10% (entry 6), whereas with complex **2**, quantitative conversion of base was still achieved, with a TON of 23,600 (entry 7). At an even lower catalyst loading of 1.4 μmol, complex **1** was essentially inactive (entry 8), whereas complex **2** achieved 64% conversion of DBU and a TON of 30,300. Thus, both catalysts are highly efficient in the hydrogenation of CO_2_ to DBU-formate with TONs > 30,000 h^–1^ for complex **2**. At lower catalyst loadings, both complexes appear to be susceptible to deactivation, but catalyst **2** appears to be more robust in that respect.

## Conclusions

We have shown that mononuclear Ru complexes with PN^3^P-R pincer-type ligands can be prepared cleanly, provided that sterically bulky R groups are used such as *tert*-butyl or TMPhos. Smaller R groups such as ^*i*^Pr and Ph lead to multiple products. Both six-coordinate complexes [RuClH(CO)(PN^3^P-R)] (**1** and **2**) react with KO^t^Bu to form the five-coordinate complexes [RuH(CO)(PN^3^P-R–H)] (**3** and **4**). In the case of R = ^t^Bu, reactivity studies with CO have shown that a dicarbonyl complex [RuH(CO)_2_(PN^3^P-^t^Bu–H)] (**5**) is formed, which shows temperature-dependent dynamic behavior in solution and which can be protonated to give the cationic complex [RuH(CO)_2_(PN^3^P-^t^Bu)]^+^ (**6**). Dihydrogen is reversibly activated by complex **3** to give the dihydride complex [RuH_2_(CO)(PN^3^P-^t^Bu)] (**7**). Attempts to use this dihydride complex as a hydride donor with other ruthenium carbonyl complexes led to unstable formyl complexes, which readily decarbonylated to give the stable cationic complex **6**. From these studies, it can be concluded that the conversion of syngas with these Ru-based PN^3^P catalysts suffers from CO poisoning of the H_2_ activation mechanism in these systems, precluding the hydrogenation of CO to take place.

The hydrogenation of CO_2_ on the other hand takes place readily, and both complexes **1** and **2** are highly active catalysts for the hydrogenation of CO_2_ to formate in the presence of the base DBU. TONs in excess of 30,000 have been achieved with complex **2**, which appears to show better stability compared to complex **1** at lower catalyst loadings. Further catalytic evaluations of these intriguing pincer complexes are underway.

## Supporting Information Available

Materials and methods, synthetic procedures, NMR spectra, and SC-XRD (PDF)
