# Supplementary material for: Hydrogen Activation with Ru-PN3P Pincer Complexes for the Conversion of C1 Feedstocks
Source: Inorg Chem. 2024 Feb 8;63(7):3393–401. doi: 10.1021/acs.inorgchem.3c04001 (PMC10880058; doi:10.1021/acs.inorgchem.3c04001)
Supplement: Supplementary file 1 — ic3c04001_si_001.pdf [file ic3c04001_si_001.pdf]

SUPPORTING INFORMATION

Hydrogen activation with Ru-PN^{3}P Pincer Complexes for the Conversion of C$_1$ Feedstocks

Matthew D. Morton,[a)] Boon Ying Tay,[b)] Justin J.Q. Mah,[b)] Andrew J.P. White,[a)] James D. Nobbs*,[b)] Martin van Meurs[b)] and George J.P. Britovsek*[a)]

a) Department of Chemistry, Imperial College London, Molecular Sciences Research Hub, White City Campus, 82 Wood Lane, W12 0BZ, United Kingdom.
b) Institute of Sustainability for Chemicals, Energy and Environment (ICSE^2), Agency for Science, Technology and Research (A*STAR), 1 Pesek Road, Jurong Island, Singapore 627833, Republic of Singapore.

Corresponding authors: g.britovsek@imperial.ac.uk, james_nobbs@isce2.a-star.edu.sg

Table of Contents

General S2
NMR spectroscopy S2
Further characterisation S2
Solvents and reagents S3
Synthesis of Ligands S3
N,N'-Bis(di-*tert*-butylphosphino)-2,6-diaminopyridine (PN^{3}P-^{t}Bu) S3
N,N'-Bis(2,2,6,6-tetramethylphosphinan-1-yl)-2,6-diaminopyridine (PN^{3}P-TMPhos) S4
Synthesis of complexes S5
[RuHCl(CO)(PN^{3}P-^{t}Bu)] (1) S5
[RuHCl(CO)(PN^{3}P-TMPhos)] (2) S5
[RuH(CO)(PN^{3}P-^{t}Bu–H)] (3) S6
[RuH(CO)(PN^{3}P-TMPhos–H)] (4) S7
[RuH(CO)$_2$(PN^{3}P-^{t}Bu–H)] (5) S7
[RuH(CO)$_2$(PN^{3}P-^{t}Bu)][B(3,5-(CF$_3$)$_2$C$_6$H$_3$)$_4$] (6) S8
[RuH$_2$(CO)(PN^{3}P -^{t}Bu)] (7) S9
[Ru(bipy)$_2$(CO)$_2$][B(C$_6$F$_5$)$_4$]$_2$ (8) S9
General procedure for the hydrogenation of CO$_2$ to formate S10
Additional Spectra S11
X-Ray Crystallography S29
References S31

General

All moisture and oxygen sensitive compounds were prepared using standard vacuum line, Schlenk and cannula techniques. A standard N_2 or Ar-filled glove box was used for any subsequent manipulation and storage of these compounds.

NMR spectroscopy

Standard ^{1}H, ^{31}P, ^{19}F, and ^{13}C NMR spectra were recorded using a Bruker 400 MHz or 500 MHz spectrometer at room temperature (RT) unless otherwise stated. ^{1}H NMR and ^{13}C NMR chemical shifts (δ) were referenced to the residual non-deuterated solvent signal and the ^{13}C signal of the deuterated solvent respectively. ^{19}F and ^{31}P NMR chemical shifts were referenced externally to $CFCl_3$ and 85% H_3PO_4 in H_2O respectively. NMR spectra for air sensitive compounds were recorded under a nitrogen or argon atmosphere. NMR spectra for samples under gas greater than atmospheric pressure were recorded using a thick walled, high-pressure tube. NMR signal multiplicities are described by the following abbreviations: s (singlet), d (doublet), t (triplet), q (quartet), m (unresolved multiplet), br (broad).

Further characterisation

Mass spectra were recorded using a Waters LCT Premier ES-ToF (ESI) or a Micromass Autospec Premier (LSIMS) spectrometer. Standard FTIR spectra were measured using a Perkin Elmer Spectrum GX spectrometer. Elemental analyses were carried out by the Elemental Analysis Service at London Metropolitan University or at the Institute of Sustainability for Chemicals, Energy and Environment (ISCE^2, A*STAR). UV-Vis spectra were measured using a Perkin-Elmer Lambda 20 spectrometer. Gas chromatography analysis was carried out using an Agilent 6890 Series system with an Agilent HP-5, 30m x 0.25 mm I.D., (5% Phenyl-methylpolysiloxane) column.

Solvents and reagents

Solvents and reagents were used as supplied unless otherwise specified. Solvents used in the reactions of oxygen and moisture sensitive compounds were dried and degassed according to standard techniques. Et_2O and THF were dried and freshly distilled from sodium benzophenone ketyl, under a nitrogen atmosphere. Toluene, pentane and hexane were dried by passage through a column containing 3Å molecular sieves and Q5 reagent (activated Cu(II)O). C_6D_5Cl was dried by stirring over CaH_2 followed by trap to trap distillation, and was stored in a glass ampoule inside a N_2 or Ar filled glovebox. $RuCl_3.3H_2O$ was donated by Johnson Matthey and used without further purification. ${}^tBu_2PCl$ was donated by Solvay (Rhodia) and used without further purification. All other chemicals were obtained commercially and used without further purification.

$[RuHCl(CO)(PPh_3)_3]$,[1] $[H(Et_2O)_2][B(3,5-(CF_3)_2C_6H_3)_4]$,[2] as well as *N*,*N'*-bis(di-*iso*-propylphosphino)-2,6-diaminopyridine,[3] *N*,*N'*-bis(diphenylphosphino)-2,6-diaminopyridine,[4] were prepared according to literature procedures. *N*,*N'*-bis(di-*tert*-butylphosphino)-2,6-diaminopyridine was prepared by a modification of the literature procedure.[3] TMPhos was prepared according to a previously reported procedure.[5]

Synthesis of Ligands

***N*,*N'*-Bis(di-*tert*-butylphosphino)-2,6-diaminopyridine (PN^{3}P-^{t}Bu)**

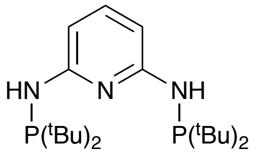

2,6-Diaminopyridine (1.000 g, 9.16 mmol) was loaded into a Schlenk flask under a nitrogen atmosphere. Dry THF (30 ml) was added by cannula and the solution cooled to −78 °C in a dry ice/acetone bath. ${}^nBuLi$ (12 mL of 1.6 M solution in hexanes, 19.2 mmol) was slowly added to this solution by syringe. A solution of ${}^tBu_2PCl$ (3.49 g, 19.3 mmol) in THF (10 mL) was added dropwise by cannula at −78 °C. The mixture was allowed to warm to RT and heated at reflux temperature overnight. After cooling to room temperature, the THF was removed under reduced pressure and DCM (30 mL) added to the light yellow residue. The suspension

was filtered through celite and then silica and the DCM removed under reduced pressure to give an off-white powder (2.72 g, 75 %).

^{1}H NMR (400 MHz, C_6D_6, δ (ppm), J (Hz)) δ 7.36 (t, $^{3}J_{HH}$ = 7.9, 1H, Ar-*H*), 6.95 (dd, $^{3}J_{HH}$ = 7.9, $^{4}J_{HH}$ = 2.4, 2H, Ar-*H*), 4.98 (br d, $^{2}J_{HP}$ = 11.4, 2H, N*H*) and 1.12 (d, $^{3}J_{HP}$ = 11.9, 36H, C(C*H*$_3$)$_3$). ^{31}P{^{1}H} NMR (162 MHz, C_6D_6, δ (ppm)) 57.0 (s). ^{13}C{^{1}H} NMR (100 MHz, $CDCl_3$, δ (ppm), J (Hz)) δ 159.9 (d, $^{2}J_{CP}$ = 21.6, *C*(Ar)), 138.9 (s, *C*(Ar)), 98.5 (d, $^{3}J_{CP}$ = 19.1, *C*(Ar)), 34.0 (d, $^{1}J_{CP}$ = 19.3, *C*(CH$_3$)$_3$) and 28.2 (d, $^{2}J_{CP}$ = 15.3, C(*C*H$_3$)$_3$). ESI/MS^{+} (*m/z*): 398.3 [M + H]$^{+}$. Anal. Calcd. for $C_{21}H_{41}N_3P_2$: C, 63.45; H, 10.40; N, 10.57. Found: C, 63.36; H, 10.29; N, 10.67.

N,N′-Bis(2,2,6,6-tetramethylphosphinan-1-yl)-2,6-diaminopyridine (PN^{3}P-TMPhos)

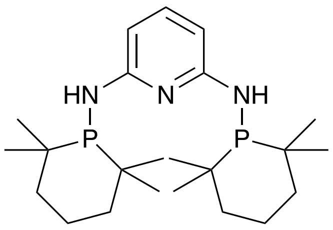

NEt_3 (0.61 mL, 4.38 mol) was added to 2,6-diaminopyridine (0.23 g, 2.1 mmol) in toluene (6 mL). The mixture was cooled to 0 °C and 1-chloro-2,2,6,6-tetramethylphosphinane was added (0.84 g, 4.38 mmol). n-BuLi (2.0M in cyclohexane, 2.19 mL, 4.38 mmol) was added after the mixture was further cooled to −78 °C. The mixture was allowed to warm to RT and then heated to 80 °C and stirred for 16 hours. After that, the reaction mixture was filtered and the volatiles were removed in vacuo. The product was recrystallised from pentane (3 mL) at −40 °C to yield a white solid. Yield = 0.42 g (47 %).

^{1}H NMR (400 MHz, $CDCl_3$, δ (ppm), J (Hz)): δ 7.26 (t, $^{3}J_{HH}$ = 8.0, 1H, Ar-*H*), 6.49 (d, $^{3}J_{HH}$ = 8.0, $^{4}J_{HP}$ = 2.2, 2H, Ar-*H*), 4.50 (br d, $^{2}J_{HP}$ = 11.1, 2H, N*H*), 1.57 (m, 8H, C*H*$_2$), 1.44 (m, 4H, C*H*$_2$), 1.15 (d, $^{3}J_{HP}$ = 17.5, 12H, C*H*$_3$) and 1.01 (d, $^{3}J_{HP}$ = 11.1, 12H, C*H*$_3$). ^{31}P{^{1}H} NMR (162 MHz, $CDCl_3$, δ (ppm), J (Hz)): δ 42.3 (s). ^{13}C{^{1}H} NMR (101 MHz, $CDCl_3$, δ (ppm), J (Hz)): δ 159.9 (d, $^{2}J_{CP}$ = 20.9, *C*(Ar)), 139.0 (t, $^{4}J_{CP}$ = 2.1, *C*(Ar)), 98.6 (d, $^{3}J_{CP}$ = 19.6, *C*(Ar)), 36.1 (s, *C*H$_2$), 31.5 (d, $^{1}J_{CP}$ = 13.1, *C$_q$*), 28.9 (d, $^{2}J_{CP}$ = 8.1, *C*H$_3$), 26.8 (d, $^{2}J_{CP}$ = 31.0, *C*H$_3$) and 20.0 (s, *C*H$_2$). HR-MS (+ve ESI): m/z (calc.) [M+H]$^{+}$ 422.2854: found 422.2854. Anal. Calcd for $C_{23}H_4N_3P$: C, 65.53; H, 9.80; N, 9.97. Found: C, 65.38; H, 9.58; N, 9.86.

Synthesis of complexes

[RuHCl(CO)(PN^{3}P-^{t}Bu)] (1)

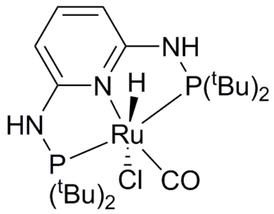

[RuHCl(CO)(PPh$_3$)$_3$] (1.00 g, 1.05 mmol) and PN^{3}P-^{t}Bu (0.50 g, 1.26 mmol) were added to a Schlenk flask under a nitrogen atmosphere and dry THF (15 mL) was added by cannula. The suspension was heated at reflux for 3 hours, resulting in an orange solution and a yellow precipitate. The THF was reduced to about 5 mL to complete the precipitation and the mixture was filtered to leave a yellow solid. This was dissolved in minimal DCM, and pentane added to precipitate a yellow powder that was washed with pentane and dried *in vacuo* (500 mg, 85 %). The complex is soluble in polar organic solvents and partially soluble in aromatic solvents.

^{1}H NMR (400 MHz, CD$_3$OD, δ (ppm), J (Hz)) δ 7.53 (t, $^3J_{HH}$ = 7.8, 1H, Ar-H), 6.50 (d, $^3J_{HH}$ = 8.0, 2H, Ar-H), 1.40 – 1.34 (m, 36H, C(CH_3)$_3$) and −24.13 (t, $^2J_{HP}$ = 18.0, 1H, RuH). ^{31}P NMR (162 MHz, CD$_3$OD, δ (ppm), J (Hz)) 134.2 (d, $^2J_{HP}$ = 18.0). ^{13}C{^{1}H} NMR (126 MHz, CD$_3$OD, δ (ppm), J (Hz)) δ 207.4 (t, $^2J_{CP}$ = 9.9, CO), 164.4 (t, $^2J_{CP}$ = 7.4, C(Ar)), 143.1 (s, C(Ar)), 99.8 (t, $^3J_{CP}$ = 3.8, C(Ar)), 41.2 (t, $^1J_{CP}$ = 9.1, C(CH$_3$)$_3$), 39.2 (t, $^1J_{CP}$ = 10.9, C(CH$_3$)$_3$), 28.9 (t, $^2J_{CP}$ = 3.3, C(CH$_3$)$_3$) and 28.6 (t, $^2J_{CP}$ = 3.3, C(CH$_3$)$_3$). IR (KBr), v(CO) 1936 (s) cm^{-1} and v(RuH) 2113 (m) cm^{-1}. LSIMS$^+$ (m/z): 528 [M − Cl]$^+$. Anal. Calcd. for $C_{22}H_{42}ClN_3OP_2Ru$: C, 46.93; H, 7.52; N, 7.46. Found: C, 46.81; H, 7.49; N, 7.59.

[RuHCl(CO)(PN^{3}P-TMPhos)] (2)

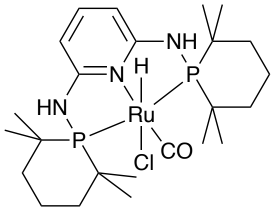

2

Carbonylchlorohydridotris(triphenylphosphine)ruthenium(II) (271 mg, 0.29 mmol) and PN^{3}P-TMPhos ligand (150 mg, 0.36 mmol) were heated at 65 °C in THF (5 mL) for 16 hours. After cooling the white precipitate was filtered, washed with Et$_2$O (2 × 5 mL) and dried in vacuo. Yield = 94 mg (56%).

^{1}H NMR (400 MHz, CD$_3$CN, δ (ppm), J (Hz)): d 8.52 (s, 2H, NH), 7.16 (t, $^3J_{HH}$ = 8.0, 1H, Ar-H), 6.72 (d, $^3J_{HH}$ = 8.0, 2H, Ar-H), 2.41 (t, $^3J_{HH}$ = 12, 2H, CH_2), 2.18 (t, $^3J_{HH}$ = 12, 2H, CH_2),

1.77 (q, $^3J_{HH}$ = 12, 2H, CH_2), 1.69 – 1.57 (m, 6H, CH_3), 1.51 (t, |$^3J_{HP}$ + $^5J_{HP}$| = 9.5, 6H, C$\underline{H_3}$), 1.28 (t, |$^3J_{HP}$ + $^5J_{HP}$| = 9.2, 6H, CH_3), 1.23 (t, |$^3J_{HP}$ + $^5J_{HP}$| = 7.0, 6H, CH_3), 1.27 (t, |$^3J_{HP}$ + $^5J_{HP}$| = 7.2, 6H, CH_3) and –14.4 (t, $^2J_{HP}$ = 20.0, 1H, Ru-*H*). ^{31}P{^{1}H} NMR (162 MHz, CD_3CN, δ (ppm)): d 123.8. ^{13}C{^{1}H} NMR (101 MHz, CD_3CN, δ (ppm), J (Hz)): δ 206.8 (t, $^2J_{CP}$ = 10.2, Ru-*C*O), 162.5 (t, $^2J_{CP}$ = 71., Ar*Cq*), 140.2 (s, Ar*C*), 99.4 (t, $^3J_{CP}$ = 3.5, Ar*C*), 39.7 (s, Cq*C*H_2), 37.7 (s, Cq*C*H_2), 37.7 (t, |$^1J_{CP}$ + $^3J_{CP}$| = 11.9, *C*(CH_3)$_2$), 37.4 (t, |$^1J_{CP}$ + $^3J_{CP}$| = 9.7, *C*(CH_3)$_2$), 29.3 (s, *C*H_3), 29.0 (s, *C*H_3) 27.2 (t, $^2J_{CP}$ = 10.1, *C*H_3), 25.8 (t, $^2J_{CP}$ = 8.4, *C*H_3) and 19.9 (s, CH_2*C*H_2CH_2). IR (KBr), $\nu_{(CO)}$ 1932 (s) cm^{-1} and ν(RuH) 2070 (m) cm^{-1}. Anal. Calcd. for $C_{24}H_{42}ClN_3OP_2Ru$: C, 49.10; H, 7.21; N, 7.16. Found: C, 48.06; H, 7.15; N, 6.72.

[RuH(CO)(PN^{3}P-^{t}Bu–H)] (3)

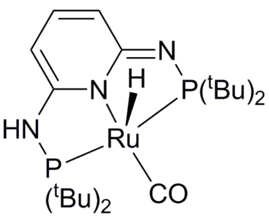

[RuHCl(CO)(PN^{3}P-^{t}Bu)] (120 mg, 0.213 mmol) and KO^{t}Bu (30 mg, 0.268 mmol) were added to a Schlenk flask in a nitrogen filled glovebox and dry THF (10 ml) was added. The suspension was stirred at RT until a red/orange solution was obtained (30 minutes) and the THF was removed under reduced pressure. The red residue was extracted with DCM (10 mL), filtered and the DCM removed under reduced pressure. The red powder was washed with pentane at −78 °C and dried *in vacuo* (70 mg, 51 %). Noteworthy, the colour of freshly prepared [RuH(CO)(PN^{3}P-^{t}Bu–H)] is red, but was reported by Huang and co-workers as a brown-red solid.[6] We observed that this complex slowly decomposes at RT under an inert atmosphere, turning from red to brown on a timescale of several months, though when stored in a freezer this decomposition is not observed.

^{1}H NMR (400 MHz, C_6D_6, δ (ppm), J (Hz)) δ 6.94 – 6.89 (m, 1H, Ar-*H*), 6.79 (d, $^3J_{HH}$ = 8.4, 1H, Ar-*H*), 5.18 (d, $^3J_{HH}$ = 7.2, 1H, Ar-*H*), 4.26 (br s, 1H, N*H*), 1.43 (d, $^3J_{HP}$ = 13.6, 9H, C(CH_3)$_3$), 1.29 (d, $^3J_{HP}$ = 12.8, 9H, C(CH_3)$_3$), 0.96 (d, $^3J_{HP}$ = 14.0, 9H, C(CH_3)$_3$), 0.89 (d, $^3J_{HP}$ = 13.6, 9H, C(CH_3)$_3$) and−25.93 (t, $^2J_{HP}$ = 16.4, 1H, Ru*H*). ^{31}P{^{1}H} NMR (162 MHz, C_6D_6, δ (ppm), J (Hz)): 130.7 (d, $^2J_{PP}$ = 220) and 128.2 (d, $^2J_{PP}$ = 220). ^{13}C{^{1}H} NMR (126 MHz, C_6D_6, δ (ppm), J (Hz)) δ 209.4 (dd, $^2J_{CP}$ = 9.8, $^2J_{CP}$ = 5.7, *C*O), 173.9 (dd, $^2J_{CP}$ = 7.8, $^3J_{CP}$ = 2.0, *C*(Ar)), 160.6 (dd, $^2J_{CP}$ = 9.5, $^3J_{CP}$ = 7.7, *C*(Ar)), 138.0 (s, *C*(Ar)), 106.5 (d, $^3J_{CP}$ = 21.1, *C*(Ar)), 84.9 (d, $^3J_{CP}$ = 7.5, *C*(Ar)), 41.6 (dd, $^1J_{CP}$ = 23.7, $^3J_{CP}$ = 3.0, *C*(CH_3)$_3$), 38.4 (d, $^1J_{CP}$ = 13.0, *C*(CH_3)$_3$), 36.3 (d, $^1J_{CP}$ = 15.9, *C*(CH_3)$_3$), 36.0 (dd, $^1J_{CP}$ = 29.1, $^3J_{CP}$ = 4.6 *C*(CH_3)$_3$), 28.3 (d, $^2J_{CP}$ = 5.0,

$C(CH_3)_3$), 27.9 (d, $^2J_{CP}$ = 4.5, $C(CH_3)_3$), 27.7 (d, $^2J_{CP}$ = 6.6, $C(CH_3)_3$) and 27.6 (d, $^2J_{CP}$ = 7.1, $C(CH_3)_3$). IR (KBr), $\nu_{(CO)}$ 1885 (s) cm^{-1} and ν(RuH) 2105 (m) cm^{-1}. ESI/MS$^+$ (*m/z*): 528 $[M + H]^+$. Anal. Calcd. for $C_{22}H_{41}N_3OP_2Ru$: C, 50.18; H, 7.85; N, 7.98. Found: C, 49.99; H, 7.97; N, 7.90.

[RuH(CO)(PN^{3}P-TMPhos–H)] (4)

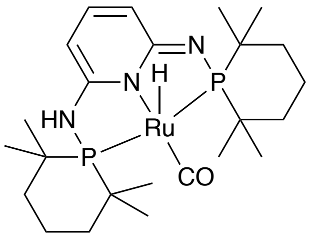

[RuHCl(CO)(PN^{3}P-TMPhos)] (**2**) (25 mg, 0.04 mmol) and KO^{t}Bu (6.0 mg, 0.051 mmol) were dissolved in MeCN (2 mL) and stirred at RT for 2 h. The solution was passed through a glass filter and the solvent removed *in vacuo* to give an off-white solid. Yield = 20 mg (80%). The complex also appears to decompose in solution gradually darkening over a few days with the formation of precipitate.

^{1}H NMR (400 MHz, CD_3CN, δ (ppm), J (Hz)) 6.83 (m, 1H, Ar*H*), 5.77 (d, $^4J_{HP}$ = 7.8, 2H, Ar*H*), 5.34 (br. s. 1H, N*H*), 2.05 – 1.68 (m, 6H, CH_2), 1.60 – 1.48 (m, 6H, CH_2), 1.46 (t, $|^1J_{CP} + {}^3J_{CP}|$ = 8.8, 6H, CH_3), 1.24 (t, $|^1J_{CP} + {}^3J_{CP}|$ = 8.5, 6H, CH_3), 1.14 (t, $|^1J_{CP} + {}^3J_{CP}|$ = 6.6, 6H, CH_3), 0.94 (t, $|^1J_{CP} + {}^3J_{CP}|$ = 7.0, 6H, CH_3) and −14.5 (t, $^2J_{HP}$ = 19.3, 1H, Ru-*H*). $^{13}C\{^1H\}$ NMR (126 MHz, CD_3CN, δ (ppm), J (Hz)) δ 208.9 (m, Ru-*C*O), 138.2 (d, $^4J_{CP}$ = 1.2, Ar*C*H), 134.4 (d, $^2J_{CP}$ = 19.7, ArC_q), 129.8 (d, $^3J_{CP}$ = 23.5, Ar*C*), 39.9 (s, C_qCH_2), 37.9 (s, C_qCH_2), 37.1 (d, $^1J_{CP}$ = 13.3, $C(CH_3)_2$), 36.9 (t, $|^1J_{CP} + {}^3J_{CP}|$ = 10.8, $C(CH_3)_2$), 29.4 (d, $^2J_{CP}$ = 9.1, CH_3), 27.2 (t, $|^2J_{CP} + {}^4J_{CP}|$ = 9.4, CH_3), 26.0 (t, $|^2J_{CP} + {}^4J_{CP}|$ = 7.8, CH_3) and 20.3 (s, $CH_2CH_2CH_2$). $^{31}P\{^1H\}$ NMR (162 MHz, CD_3CN, δ (ppm), J (Hz)) δ 122.1 (br. s).

[RuH(CO)$_2$(PN^{3}P-^{t}Bu–H)] (5)

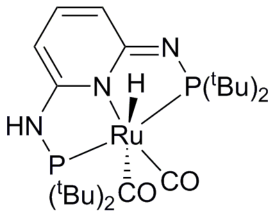

$[Ru_3(CO)_{12}]$ (100 mg, 0.16 mmol) and PN^{3}P(^{t}Bu) (187 mg, 0.47 mmol) were added to a Schlenk flask and toluene (5 mL) was then added. The orange/yellow suspension was heated at reflux for 17 h and cooled to RT. Pentane (10 mL) was added to the resulting suspension to complete precipitation of the pink/orange powder that was isolated by filtration, washed with pentane and dried *in vacuo*. (172 mg, 66 %).

^{1}H NMR (400 MHz, CD_3OD, δ (ppm), J (Hz)) δ 7.30 (t, $^{3}J_{HH}$ = 8.3, 1H, Ar-*H*), 6.26 (d, $^{3}J_{HH}$ = 8.3, 2H, Ar-*H*), 1.52 (m, 18H, C(C*H*$_3$)$_3$), 1.39 (m, 18H, C(C*H*$_3$)$_3$) and −6.34 (t, $^{2}J_{HP}$ = 19.0, 1H, Ru*H*). $^{31}P\{^{1}H\}$ NMR (162 MHz, CD_3OD, δ (ppm) 141.2 (s). $^{13}C\{^{1}H\}$ NMR (101 MHz, CD_3OD, δ (ppm), J (Hz)) δ 202.8 (m, *C*O), 200.6 (m, *C*O), 164.2 – 163.5 (m, *C*(Ar)), 141.5 – 140.5 (m, *C*(Ar)), 100.0 – 99.1 (m, *C*(Ar)), 41.7 (m, *C*(CH$_3$)$_3$), 40.1 (m, *C*(CH$_3$)$_3$), 30.1 (s, C(*C*H$_3$)$_3$) and 28.8 (s, C(*C*H$_3$)$_3$). IR (KBr), $\nu_{(CO)}$ 1989 (s), 1940 (s) cm^{-1} and ν(RuH) 2044 (m) cm^{-1}. ESI/MS^{+} (*m/z*): 528 $[M-CO+H]^{+}$ and 556 $[M+H]^{+}$. Anal. Calcd. for $C_{23}H_{41}N_3O_2P_2Ru$: C, 49.81; H, 7.45; N, 7.58. Found: C, 49.89; H, 7.32; N, 7.50.

[RuH(CO)$_2$(PN^{3}P-^{t}Bu)][B(3,5-(CF$_3$)$_2$C$_6$H$_3$)$_4$] (6)

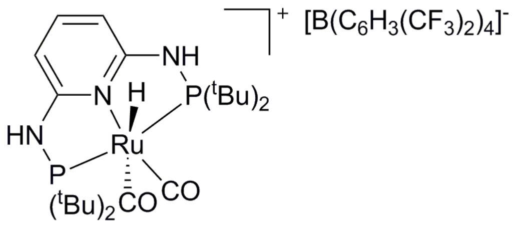

[RuH(CO)$_2$(PN^{3}P-^{t}Bu)] (50 mg, 0.09 mmol) and [H(Et$_2$O)$_2$][B(3,5-(CF$_3$)$_2$C$_6$H$_3$)$_4$] (95 mg, 0.09 mmol) were added to a Schlenk flask in a nitrogen filled glovebox and dry THF (5 mL) was added. The colourless solution was stirred at RT for 30 minutes, the THF was reduced to about 0.5 mL and Et_2O added to precipitate a cream powder. The crude product was dissolved in minimal DCM, precipitated and washed with pentane, dried *in vacuo* to give the pure product. (99 mg, 78 %). Crystals suitable for Single Crystal X-ray diffraction were obtained by diffusion of hexane into a benzene solution of the complex.

^{1}H NMR (400 MHz, C_6D_5Cl, δ (ppm), J (Hz)) δ 8.41 (br s, 8H, Ar-*H*), 7.76 (br s, 4H, Ar-*H*), 7.06 (t, $^{3}J_{HH}$ = 8.1, 1H, Ar-*H*), 6.10 (d, $^{3}J_{HH}$ = 8.1, 2H, Ar-*H*), 5.27 (br s, 2H, N*H*), 1.33 – 1.25 (m, 18H, C(C*H*$_3$)$_3$), 1.23 – 1.14 (m, 18H, C(C*H*$_3$)$_3$) and −6.62 (t, $^{2}J_{HP}$ = 19.6, 1H, Ru*H*). $^{31}P\{^{1}H\}$ NMR (162 MHz, C_6D_5Cl, δ (ppm), J (Hz)) δ 142.7 (s). ^{19}F NMR (377 MHz, C_6D_5Cl, δ (ppm), J (Hz)) δ −62.33 (s).$^{13}C\{^{1}H\}$ NMR (101 MHz, C_6D_5Cl, δ (ppm), J (Hz)) δ 198.5 (m, *C*O), 196.5 (m, *C*O), 162.4 (q, $^{1}J_{CB}$ = 49.8, *C*(Ar)), 159.0 (t, $^{2}J_{CP}$ = 5.6, *C*(Ar)), 141.4, 135.2, 133.9, 127.7, 125.5, 123.8, 121.7, 117.8 (m, *C*(Ar)), 100.0, 40.6 (t, $^{1}J_{CP}$ = 9.9, *C*(CH$_3$)$_3$), 38.9 (t, $^{1}J_{CP}$ = 8.9, *C*(CH$_3$)$_3$), 28.6 and 27.6. IR (KBr), $\nu_{(CO)}$ 2017, 1986 cm^{-1}, $\nu_{(RuH)}$ 2080 cm^{-1}. ESI/MS^{+} (*m/z*): 528.2 $[M-CO]^{+}$, 556.2 $[M]^{+}$. Anal. Calcd. for $C_{55}H_{54}BF_{24}N_3O_2P_2Ru$: C, 46.56; H, 3.84; N, 2.96. Found: C, 46.42; H, 3.82; N, 3.09.

[RuH_2(CO)(PN^3P -^{t}Bu)] (7)

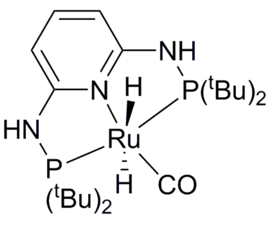

[RuH(CO)(PN^3P(^{t}Bu))] (10 mg, 0.019 mmol) was dissolved in C_6D_6 (0.5 mL) and added to a high pressure NMR tube in a N_2 filled glovebox. The tube was freeze-thaw degassed and H_2 (6 bar) added to the tube. [RuH_2(CO)(PN^3P-^{t}Bu)] was observed by ^{1}H and ^{31}P NMR spectroscopy, with *ca.* 10 % conversion of [Ru(PN^3P(^{t}Bu))H(CO)] after 4 days at RT.

^{1}H NMR (400 MHz, C_6D_6, δ (ppm), J (Hz)) δ 6.70 (t, $^3J_{HH}$ = 7.9, 1H, Ar-*H*), 5.42 (d, $^3J_{HH}$ = 7.9, 2H, Ar-*H*), 4.63 (br s, 2H, N*H*), 1.49 – 1.41 (m, 36H, C(CH_3)$_3$) and −5.34 (t, $^2J_{HP}$ = 18.7, 2H, Ru*H*). ^{31}P{^{1}H} NMR (162 MHz, C_6D_6, δ (ppm)) 156.2 (s).

[Ru(bipy)$_2$(CO)$_2$][B(C_6F_5)$_4$]$_2$ (8)

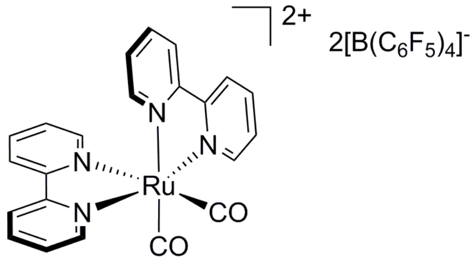

A 100 mL Parr reactor with a glass liner was charged with [$RuCl_2$(bipy)$_2$] (500 mg, 1.03 mmol) and H_2O (20 mL), then sealed, flushed (3 × 8 bar) and pressurised with CO (20 bar). The reactor was heated at 140 °C for 18 h in a graphite bath. After cooling to RT and venting the pressure, the resultant yellow solution was transferred to a 100 mL RBF and a solution of dimethylanilinium tetrakis(pentafluorophenyl)borate (1.65 g, 2.06 mmol) in DCM (15 mL) was added. The biphasic mixture was vigorously stirred until the yellow colour transferred to the organic layer, the organic layer was separated and the aqueous fraction extracted with DCM (3 × 15 ml). The combined DCM fractions were washed with H_2O (2 × 20 ml), dried over Na_2SO_4, filtered and the solvent removed under reduced pressure to give a yellow solid. This was washed with $CHCl_3$ (4 × 20 mL), Et_2O (3 × 20 mL) and hexane (3 × 20 mL) then dried *in vacuo* at 40 °C for 18 h (1.1 g, 58 %).

^{1}H NMR (400 MHz, CD_2Cl_2, δ (ppm), J (Hz)) δ 9.06 (d, $^3J_{HH}$ = 5.6, 2H, Ar-*H*), 8.55 – 8.50 (m, 4H, Ar-*H*), 8.41 (d, $^3J_{HH}$ = 8.1, 2H, Ar-*H*), 8.26 (t, $^3J_{HH}$ = 8.4, 2H, Ar-*H*), 8.03 – 7.98 (m, 2H, Ar-*H*), 7.56 – 7.51 (m, 2H, Ar-*H*) and 7.36 (d, $^3J_{HH}$ = 5.4, 2H, Ar-*H*). ^{13}C{^{1}H} NMR (126 MHz, CD_2Cl_2, δ (ppm), J (Hz)) δ 189.3 (s, *C*O), 156.0 (s, *C*(Ar)), 155.5 (s, *C*(Ar)), 154.6 (s, *C*(Ar)),

148.6 (s, *C*(Ar)), 148.4 (d, $^{1}J_{CF}$ = 239.8, *C*(Ar)), 143.4 (s, *C*(Ar)), 138.5 (d, $^{1}J_{CF}$ = 243.8, *C*(Ar)), 136.6 (d, $^{1}J_{CF}$ = 241.2, *C*(Ar)), 131.1 (s, *C*(Ar)), 130.0 (s, *C*(Ar)), 126.3 (s, *C*(Ar)), 125.7 (s, *C*(Ar)) and125.1 – 123.4 (m, *C*(Ar)).^{19}F NMR (377 MHz, C_6D_5Cl, δ (ppm), J (Hz)) δ −132.88 – −133.08 (m, 8F, *F*(Ar)), −163.24 (t, $^{3}J_{FF}$ = 20.3, 4F, *F*(Ar)), −167.20 – −167.40 (m, 8F, *F*(Ar)).IR (KBr) $\nu_{(CO)}$, 2103 (s), 2055 (m) cm^{-1}. ESI/MS^{+} (*m/z*): 1049.0 $[M-(B(C_6F_5)_4)]^+$, 235.0 $[M-2(B(C_6F_5)_4)]^{2+}$. Anal. Calcd. for $C_{24}H_{14}N_4O_8Ru_2$: C, 46.00; H, 0.88; N, 3.07. Found: C, 45.86; H, 0.81; N, 3.14.

General procedure for the hydrogenation of CO_2 to formate

A 300 mL stainless steel PARR reactor was evacuated at 140 °C for 3 h. After this time is was allowed to cool to RT and cycled 3 times with argon/vacuum. Under a stream of argon, DMF (30 mL), catalyst solution (5 mL) and DBU (10 mL) were introduced to the reactor. Mechanical stirring was started (1000 rpm) and the vessel was heated to 90 °C. At this temperature CO_2/H_2 (7.5:7.5 bar) was introduced into the reactor from a gas reservoir. The pressure in the reaction vessel was maintained throughout the reaction. After 1 h the reaction was cooled to RT and the reaction vessel vented. An accurate amount of internal standard (mesitylene) was added and the liquid fraction analysed by ^{1}H NMR spectroscopy.

Additional Spectra

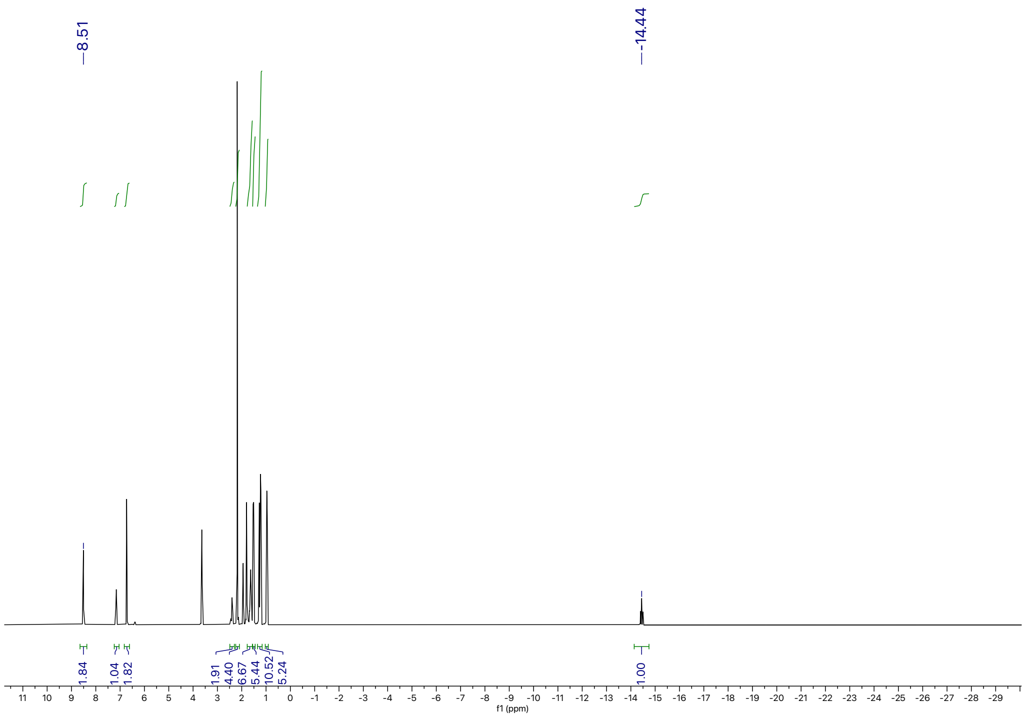

Figure S1. ^{1}H NMR spectrum of complex [RuHCl(CO)(PN^{3}P-TMPhos)] (**2**) in CD_3CN at 298K.

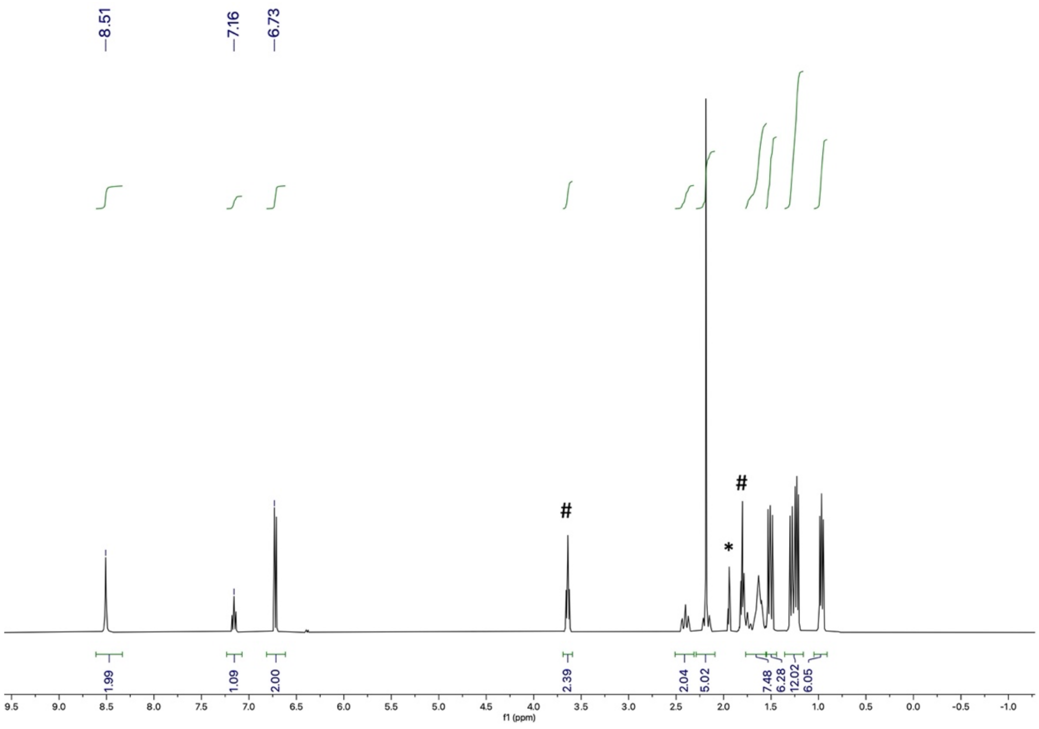

Figure S2. Expanded ^{1}H NMR spectrum of complex [RuHCl(CO)(PN^{3}P-TMPhos)] (**2**) in CD_3CN (*) at 298K (hydride excluded). (# = residual THF).

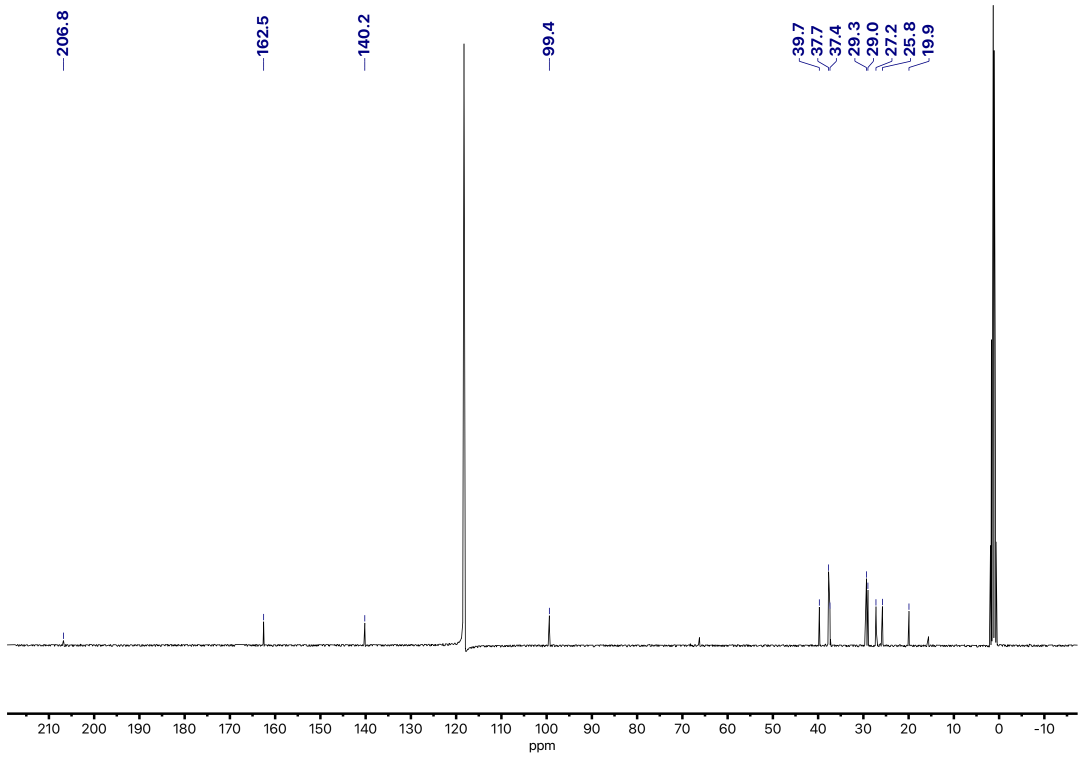

Figure S3. $^{13}C\{^{1}H\}$ NMR spectrum of complex [RuHCl(CO)(PN^{3}P-TMPhos)] (**2**) in CD_3CN at 298K.

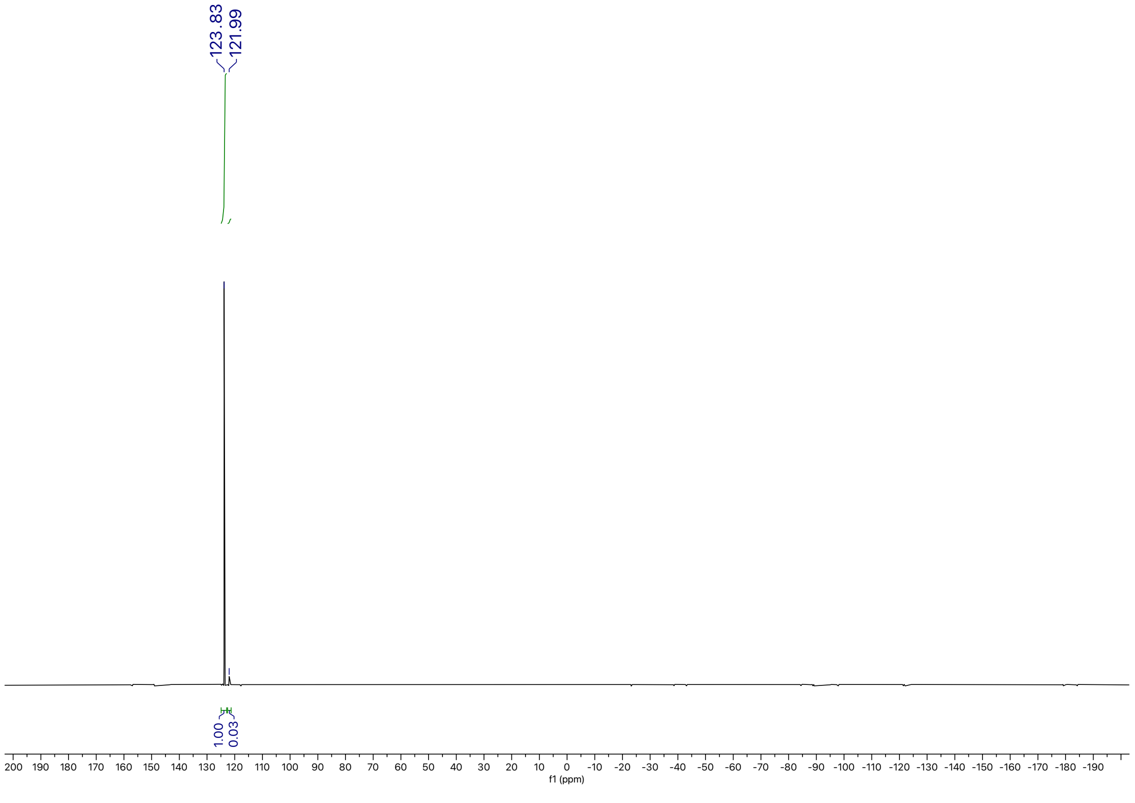

Figure S4. $^{31}P\{^{1}H\}$ NMR spectrum of complex [RuHCl(CO)(PN^{3}P-TMPhos)] (**2**) in CD_3CN at 298K.

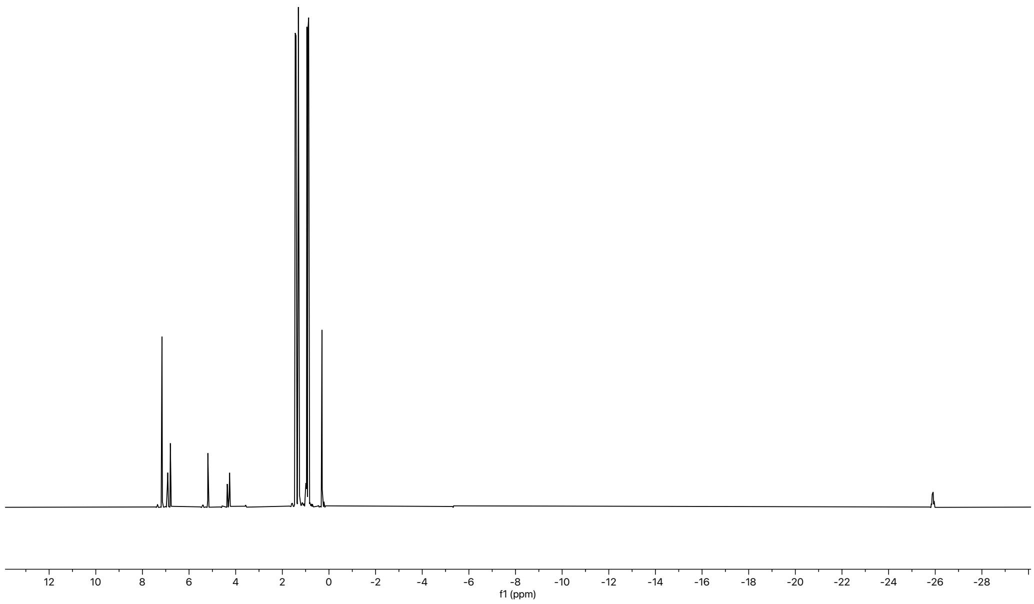

Figure S5. ^1H NMR spectrum of complex [RuH(CO)(PN^{3}P-^{t}Bu-H)] (**3**) in d_6-benzene at 298K.

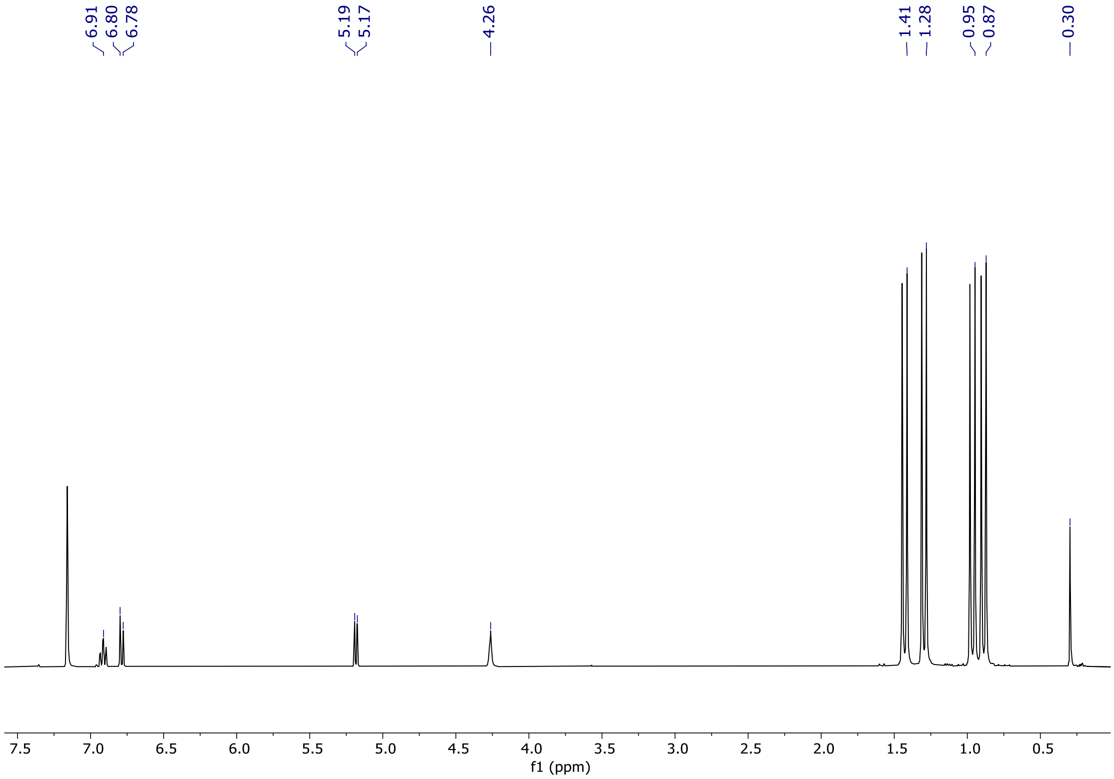

Figure S6. Expanded ^1H NMR spectrum of complex [RuH(CO)(PN^{3}P-^{t}Bu-H)] (**3**) in d_6-benzene at 298K (hydride excluded).

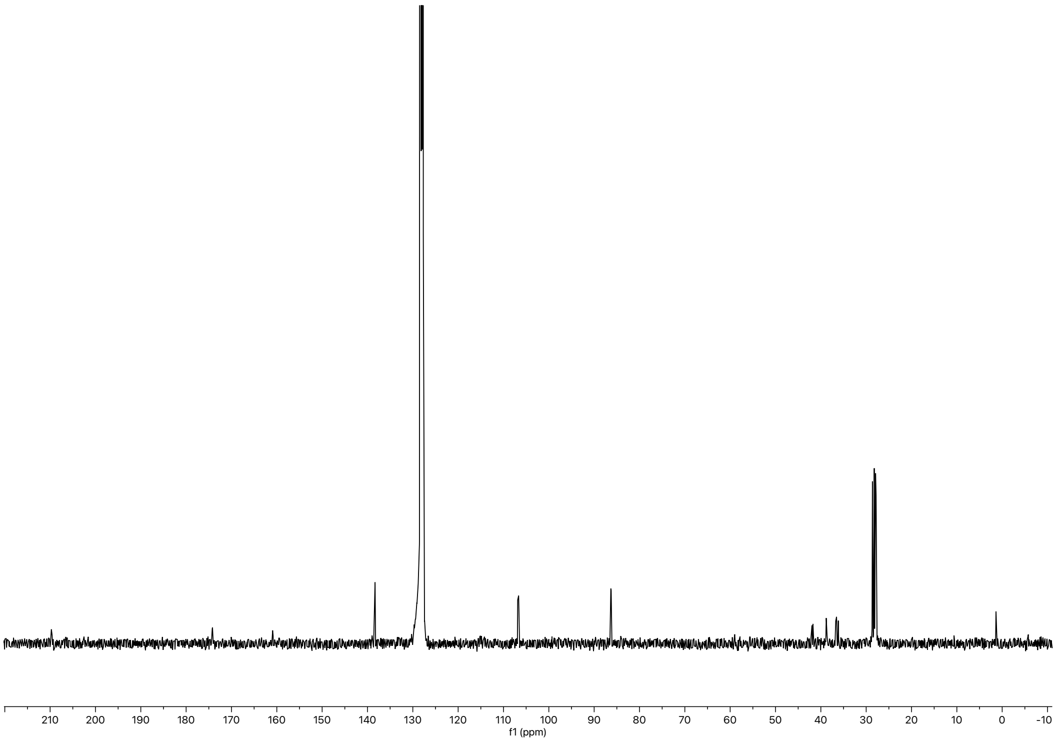

Figure S7. $^{13}C\{^1H\}$ NMR spectrum of complex [RuH(CO)(PN^{3}P-^{t}Bu-H)] (**3**) in d_6-benzene at 298K.

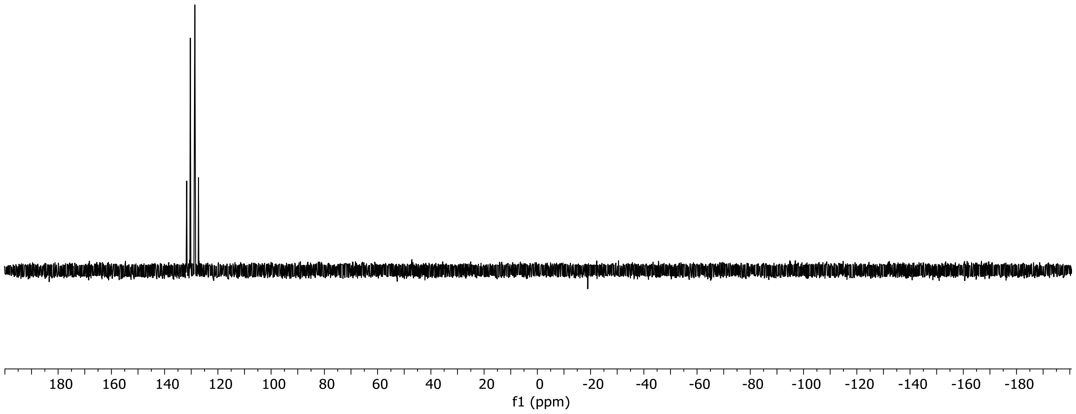

Figure S8. $^{31}P\{^1H\}$ NMR spectrum of complex [RuH(CO)(PN^{3}P-^{t}Bu-H)] (**3**) in d_6-benzene at 298K.

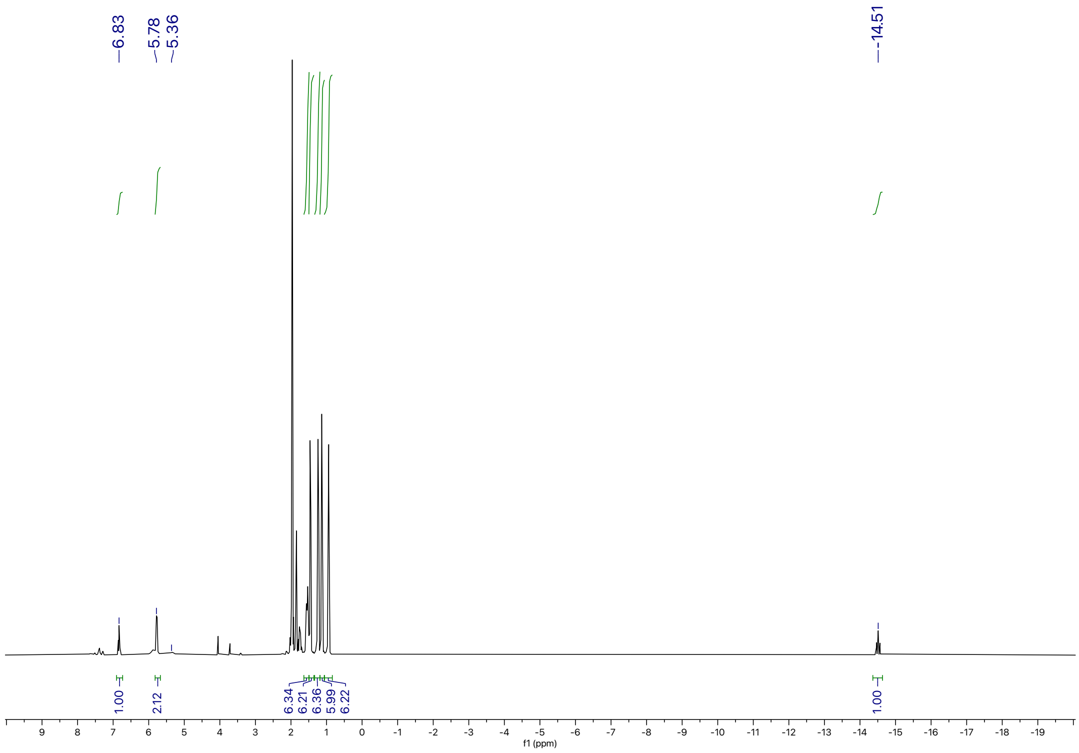

Figure S9. ^{1}H NMR spectrum of complex [RuH(CO)(PN^{3}P-TMPhos–H)] (**4**) in CD_3CN at 298K.

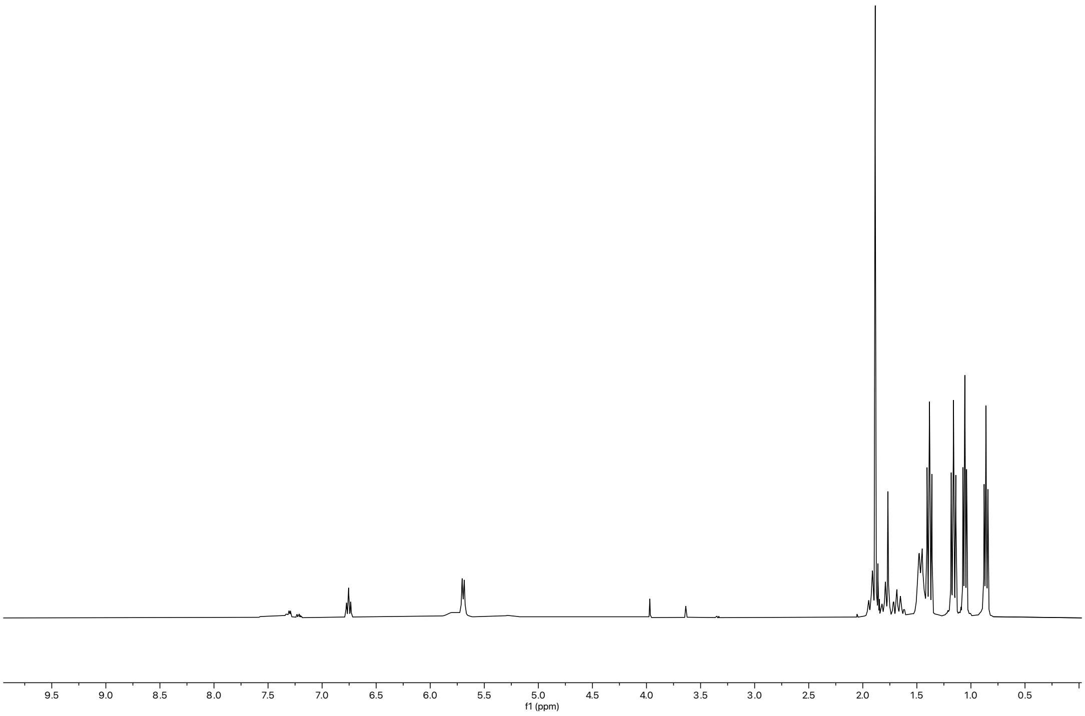

Figure S10. Expanded ^{1}H NMR spectrum of complex [RuH(CO)(PN^{3}P-TMPhos–H)] (**4**) in CD_3CN at 298K (hydride excluded).

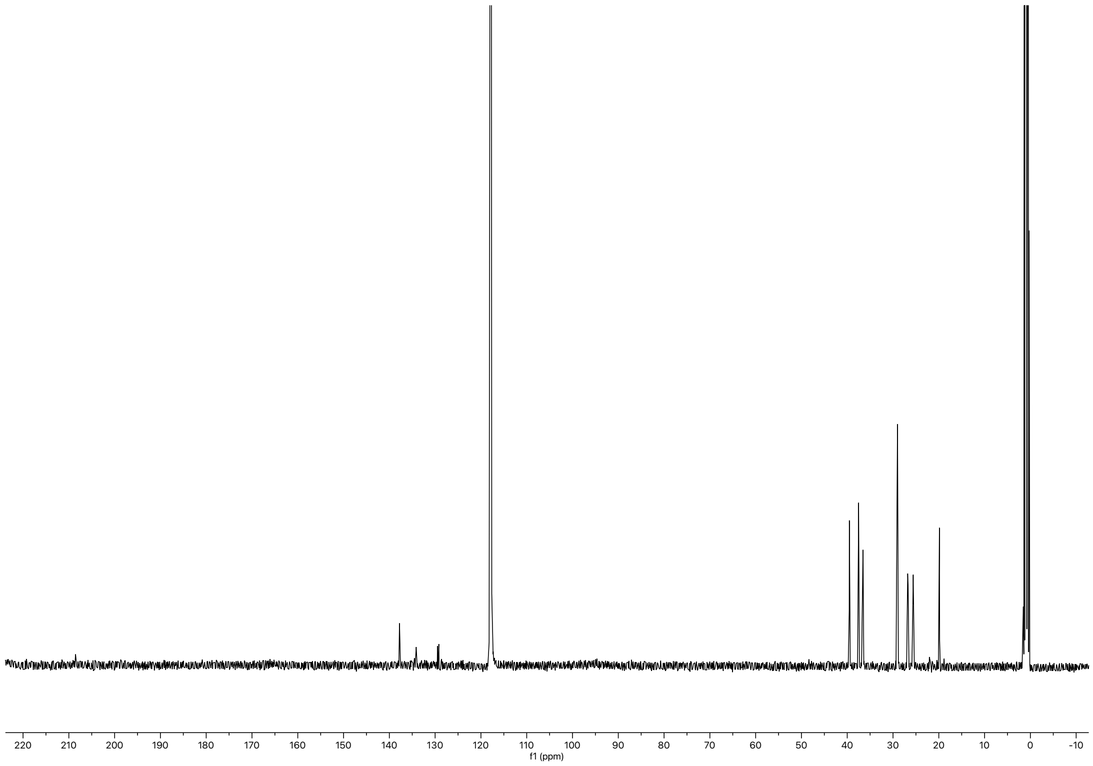

Figure S11. $^{13}C\{^{1}H\}$ NMR spectrum of complex [RuH(CO)(PN^{3}P-TMPhos–H)] (**4**) in CD_3CN at 298K.

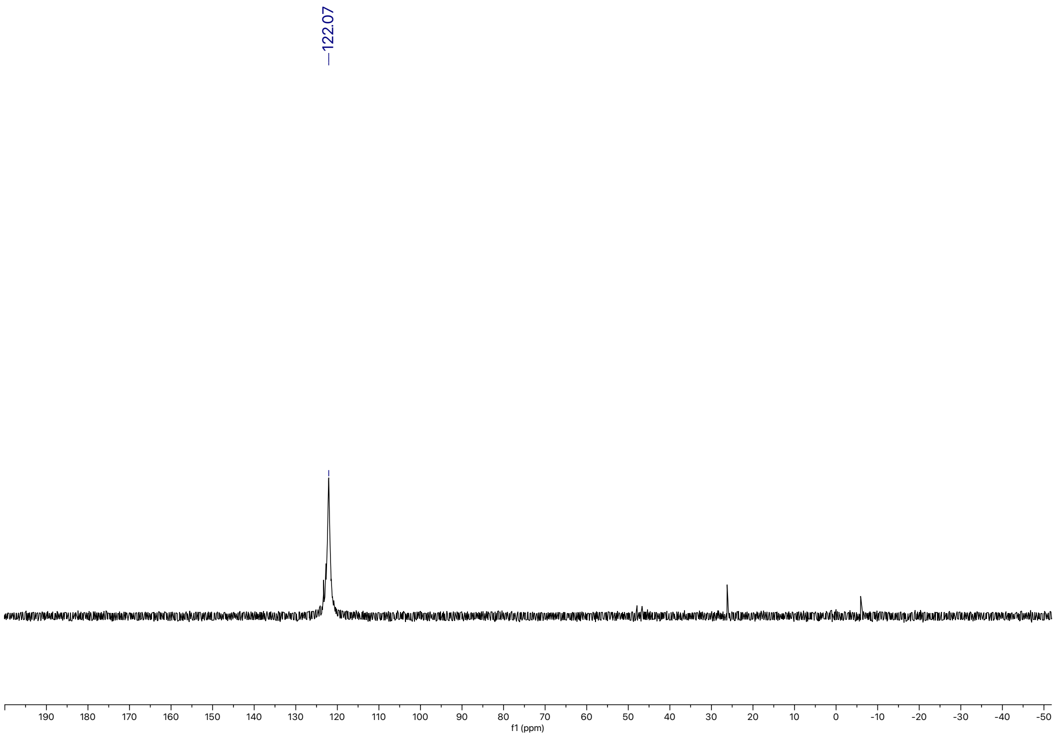

Figure S12. $^{31}P\{^{1}H\}$ NMR spectrum of complex [RuH(CO)(PN^{3}P-TMPhos–H)] (**4**) in CD_3CN at 298K.

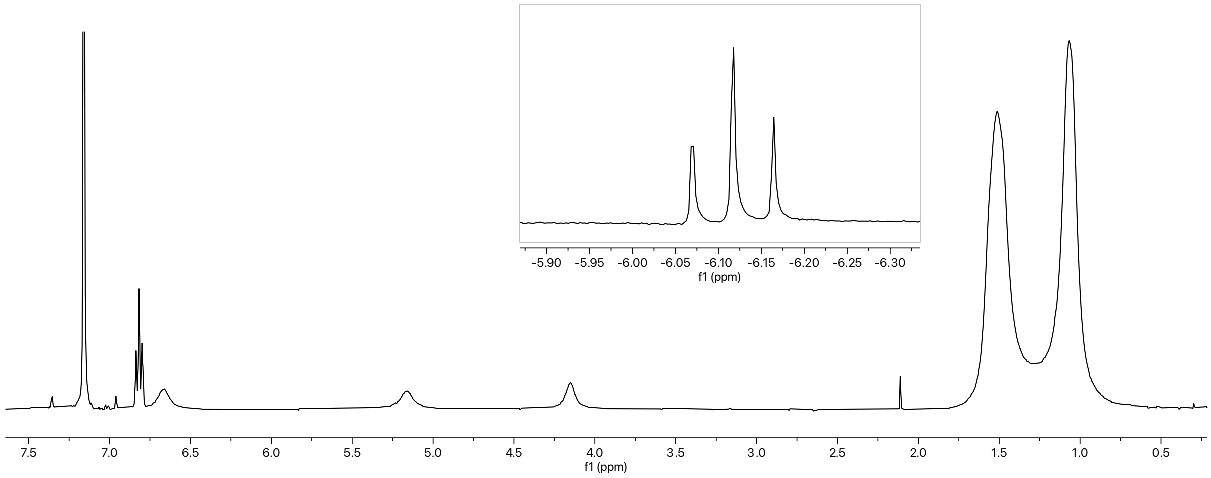

Figure S13. ^{1}H NMR spectrum of complex [RuH(CO)$_2$(PN^{3}P-^{t}Bu-H)] (**5**) in d_6-benzene at 298K.

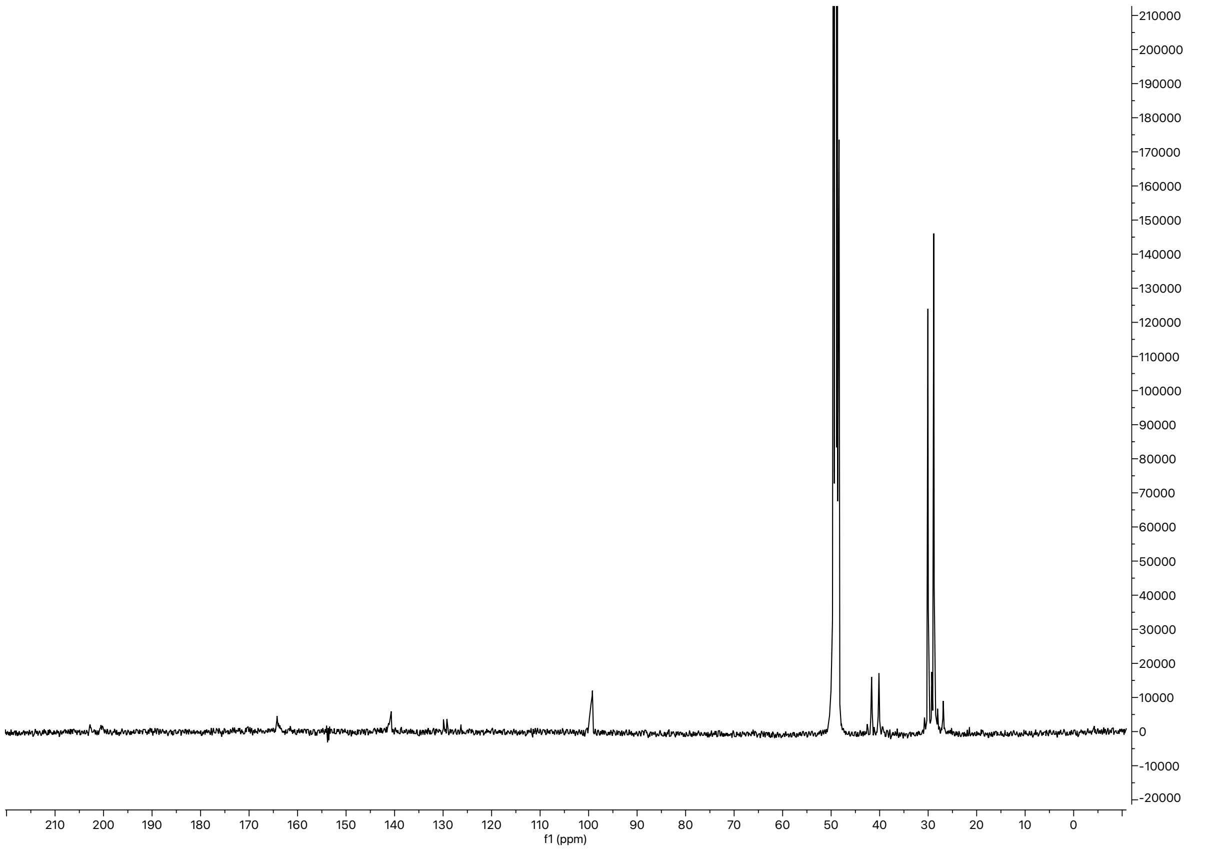

Figure S14. $^{13}C\{^{1}H\}$ NMR spectrum of complex [RuH(CO)$_2$(PN^{3}P-^{t}Bu-H)] (**5**) in d_4-Methanol at 298K.

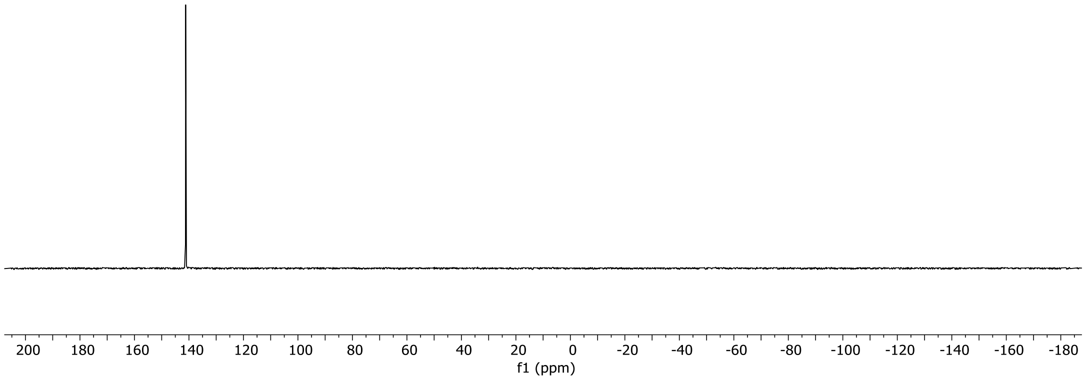

Figure S15. $^{31}P\{^{1}H\}$ NMR spectrum of complex $[RuH(CO)_2(PN^3P\text{-}^{t}Bu\text{-}H)]$ (**5**) in d_4-Methanol at 298K.

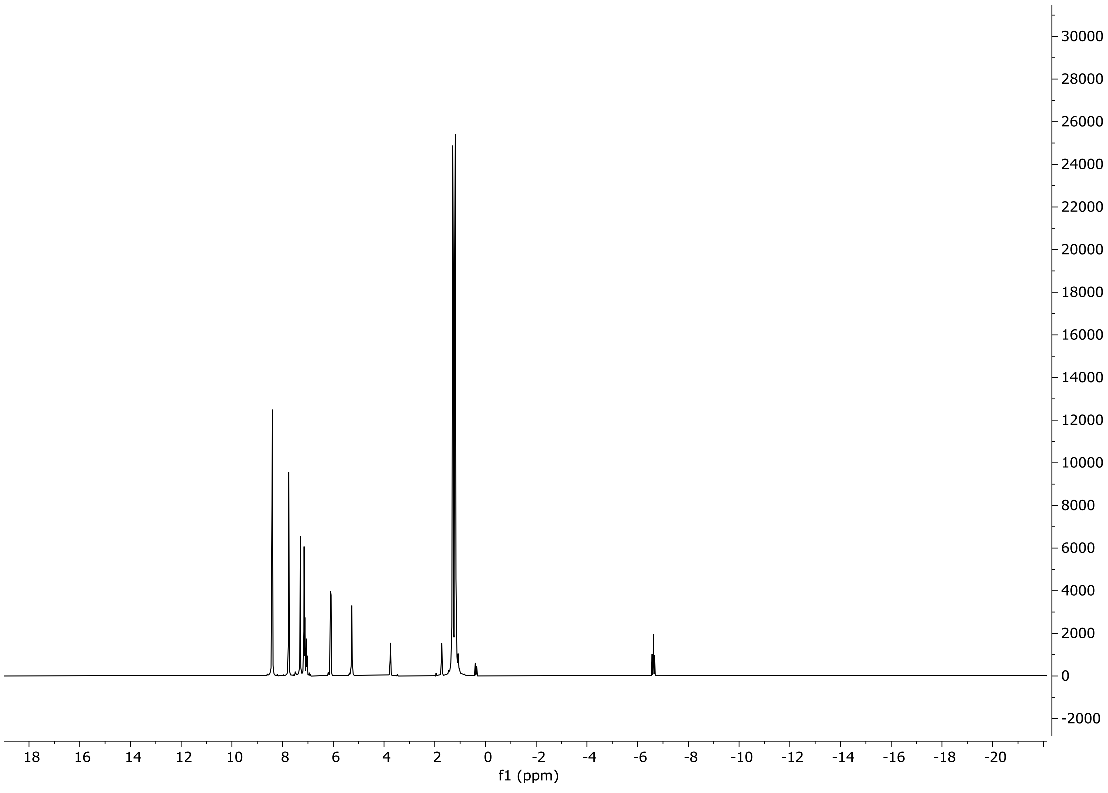

Figure S16. ^{1}H NMR spectrum of complex $[RuH(CO)_2(PN^3P\text{-}^{t}Bu)][B(C_6H_3(CF_3)_2)_4]$ (**6**) in C_6D_5Cl at 298K.

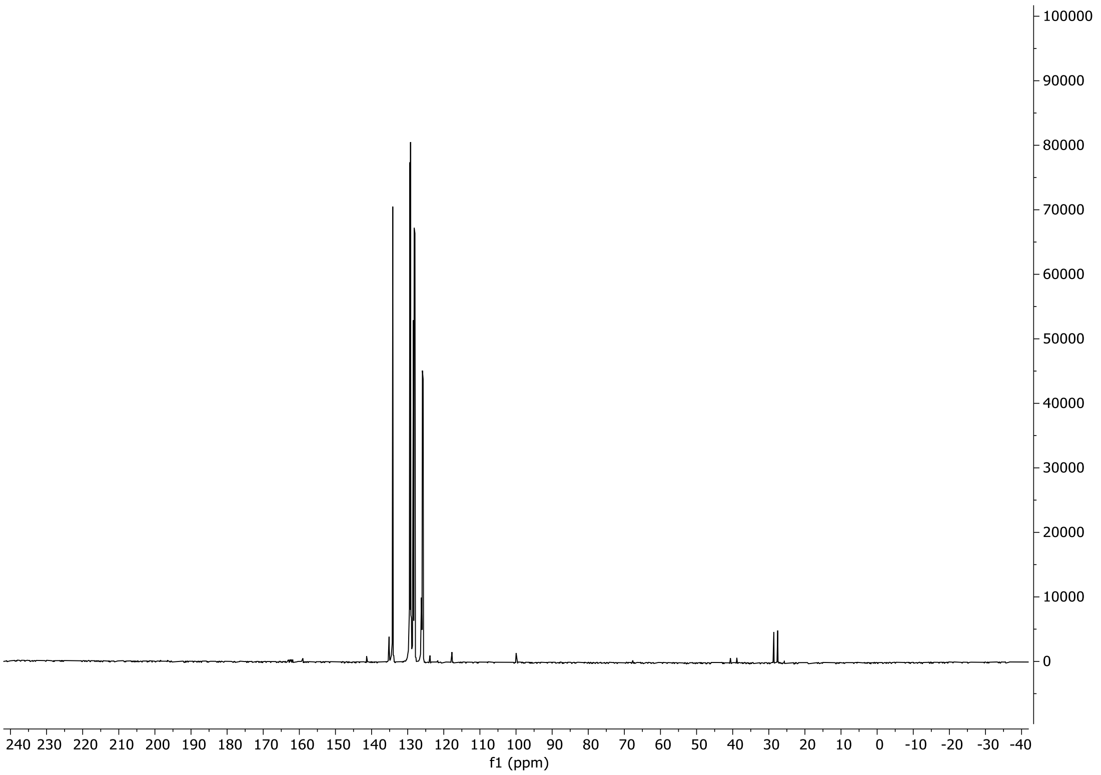

Figure S17. $^{13}C\{^{1}H\}$ NMR spectrum of complex $[RuH(CO)_2(PN^3P\text{-}^tBu)][B(C_6H_3(CF_3)_2)_4]$ (**6**) in C_6D_5Cl at 298K.

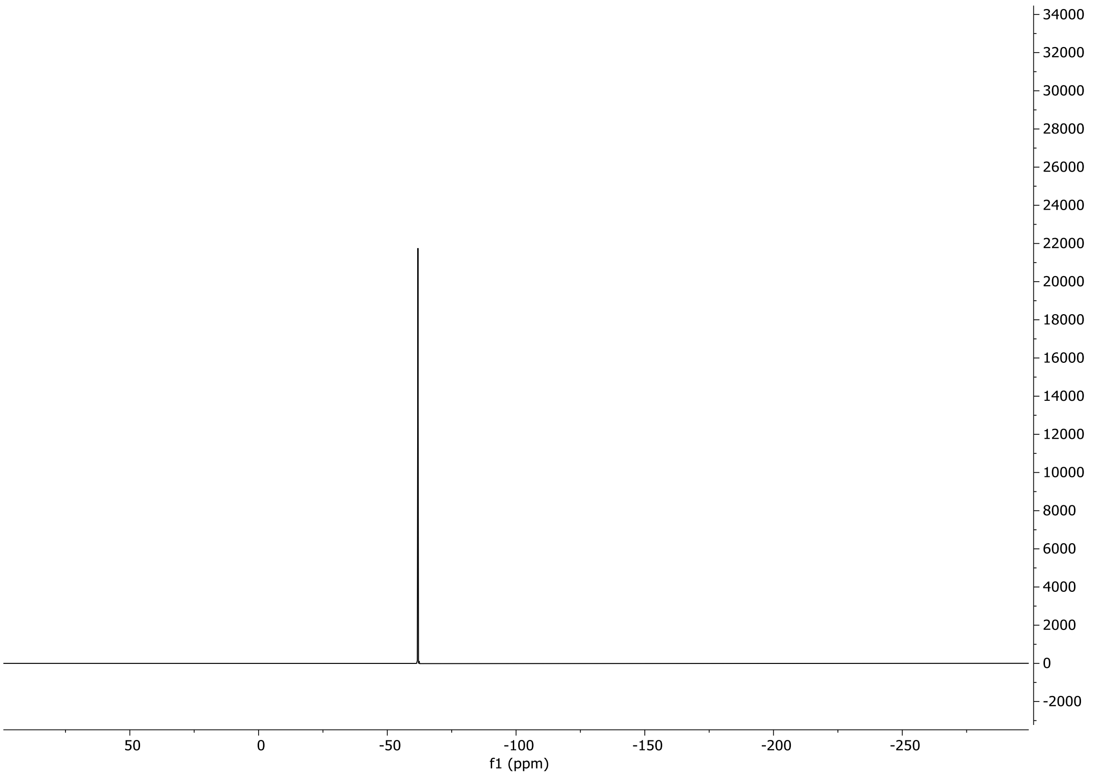

Figure S18. $^{31}P\{^{1}H\}$ NMR spectrum of complex $[RuH(CO)_2(PN^3P\text{-}^tBu)][B(C_6H_3(CF_3)_2)_4]$ (**6**) in C_6D_5Cl at 298K.

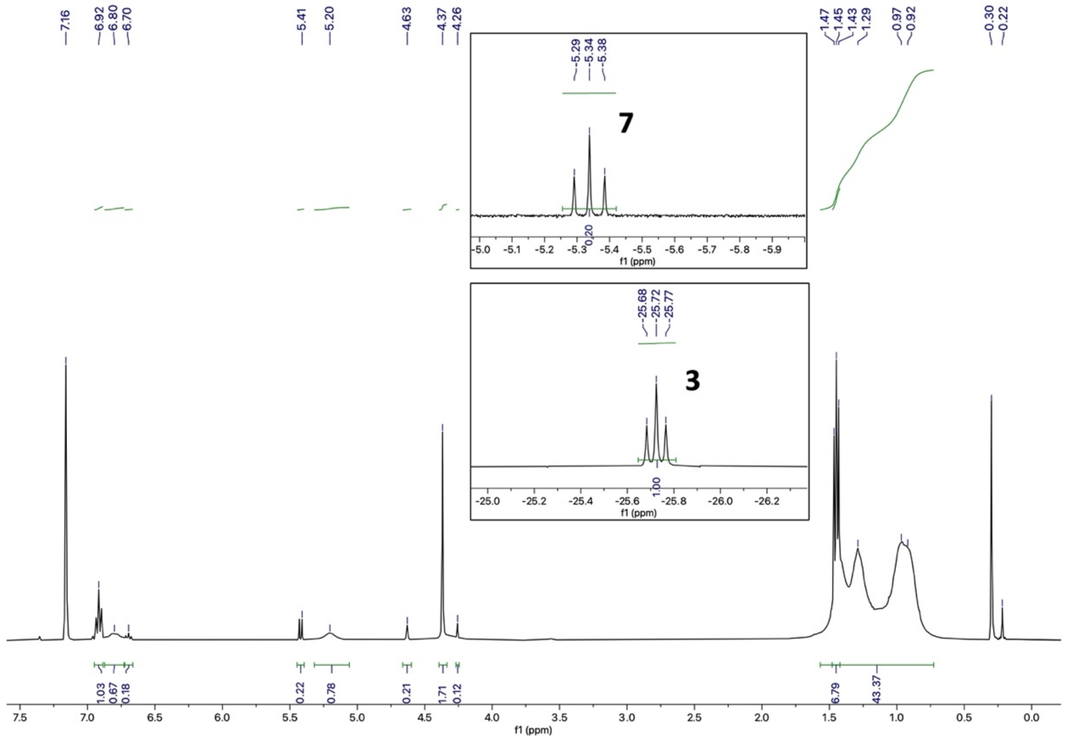

Figure S19. ^{1}H NMR spectrum of the reaction between [RuH(CO)(PN^{3}P-^{t}Bu-H)] (**3**) and H_2 (5 bar) to form [RuH$_2$(CO)(PN^{3}P-^{t}Bu)] (**7**) (~10%) in d_6-benzene at 298 K after 4 days.

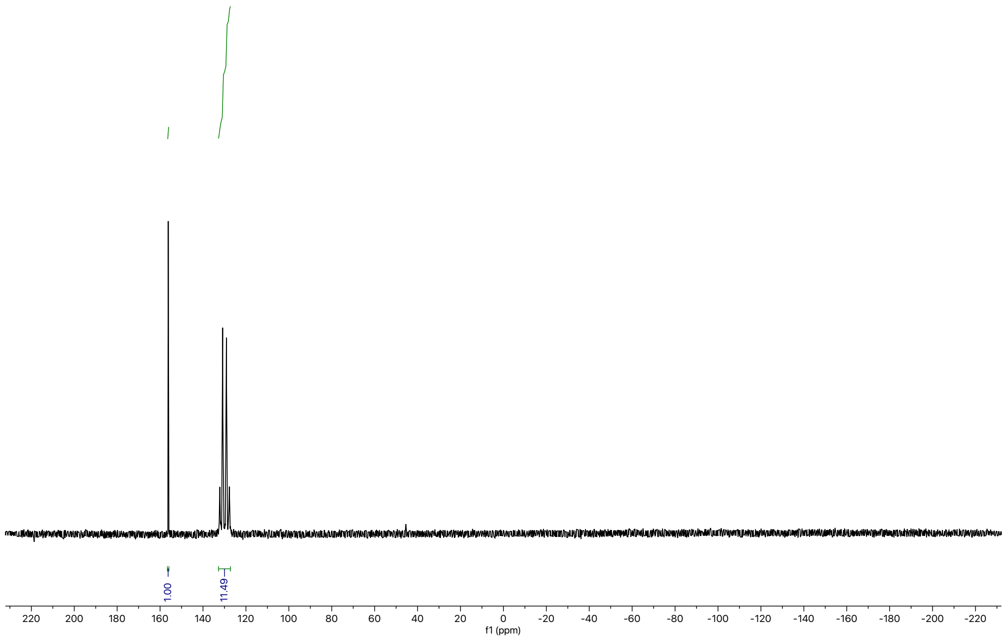

Figure S20. ^{31}P NMR spectrum of the reaction between [RuH(CO)(PN^{3}P-^{t}Bu-H)] (**3**) and H_2 (5 bar) to form [RuH$_2$(CO)(PN^{3}P-^{t}Bu)] (**7**) (~10%) in d_6-benzene at 298 K after 4 days.

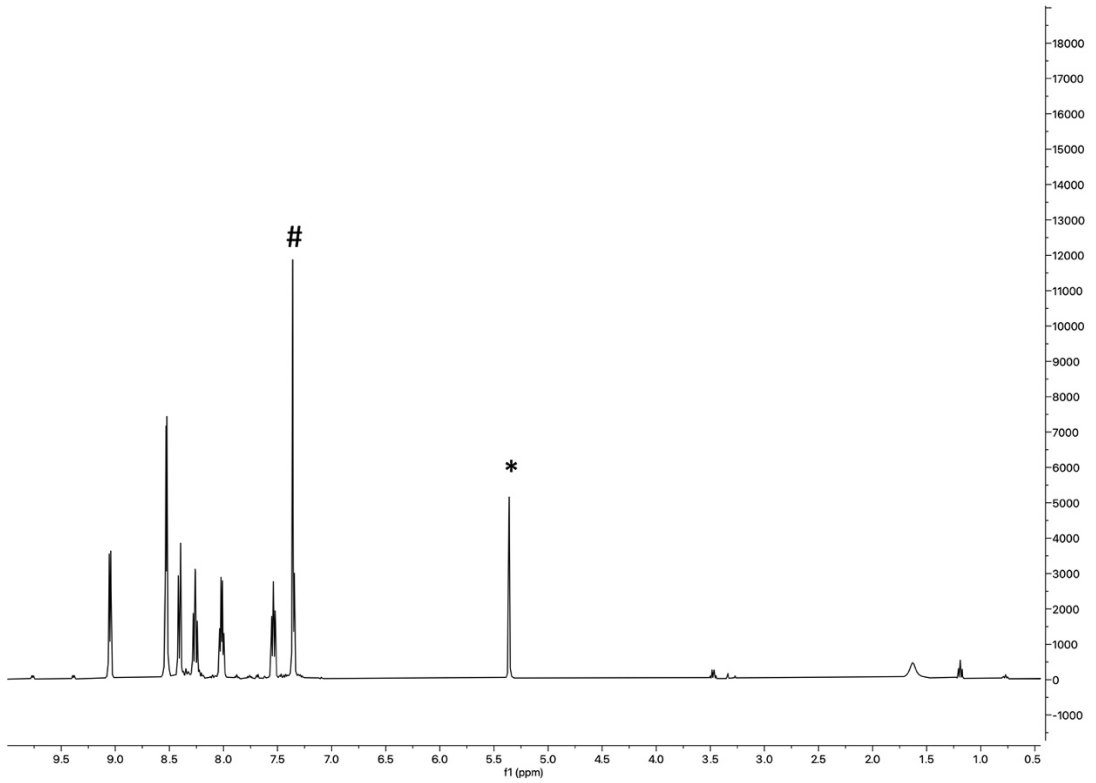

Figure S21. ^{1}H NMR spectrum of complex $[Ru(bipy)_2(CO)_2](B(C_6F_5)_4)_2$ (**8**) in CD_2Cl_2 (*) at 298 K. (# = residual $CHCl_3$).

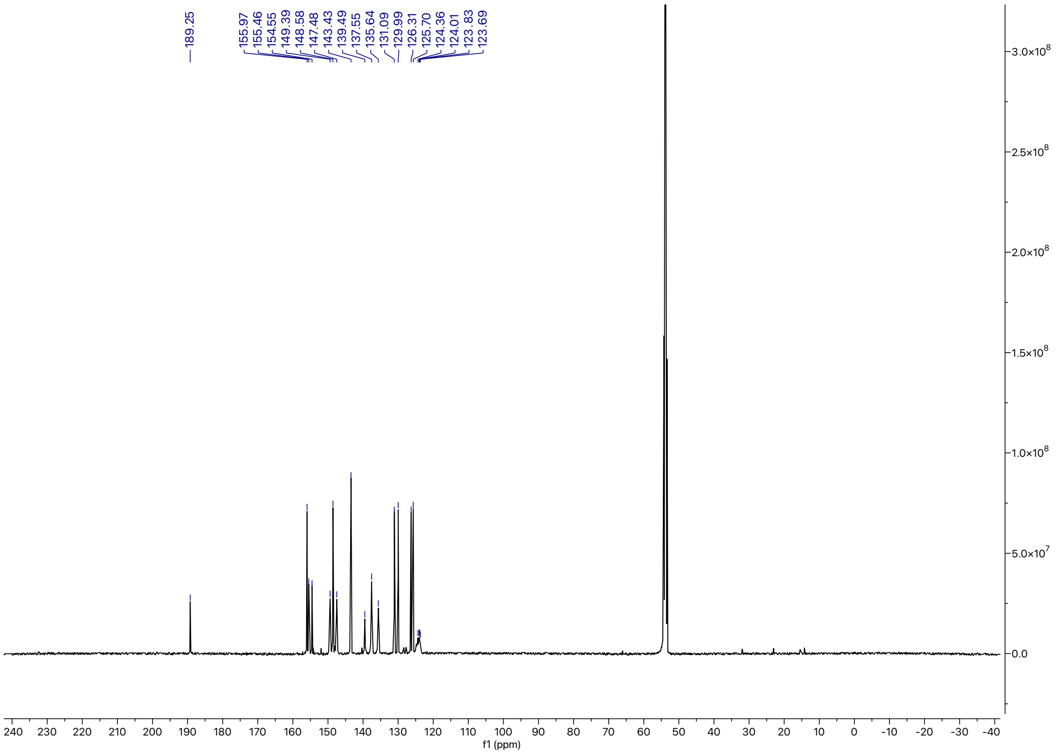

Figure S22. $^{13}C\{^{1}H)$ NMR spectrum of complex $[Ru(bipy)_2(CO)_2](B(C_6F_5)_4)_2$ (**8**) in CD_2Cl_2 at 298 K.

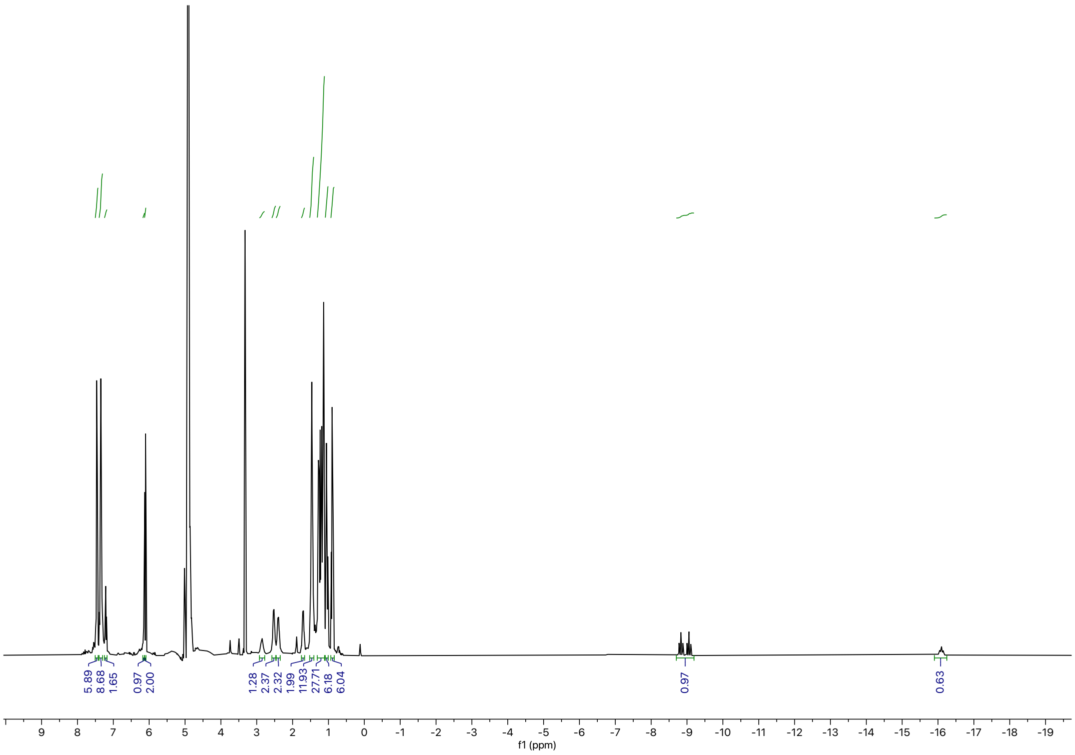

Figure S23. ^{1}H NMR spectrum of the product obtained from the reaction between PN^{3}P-^{i}Pr and [Ru(PPh$_3$)$_3$HCl(CO)] in d_4-methanol at 298K. Assumed to be a mixture of [Ru(PN^{3}P-^{i}Pr)(PPh$_3$)H(CO)]Cl and [Ru(PN^{3}P-^{i}Pr)HCl(CO)].

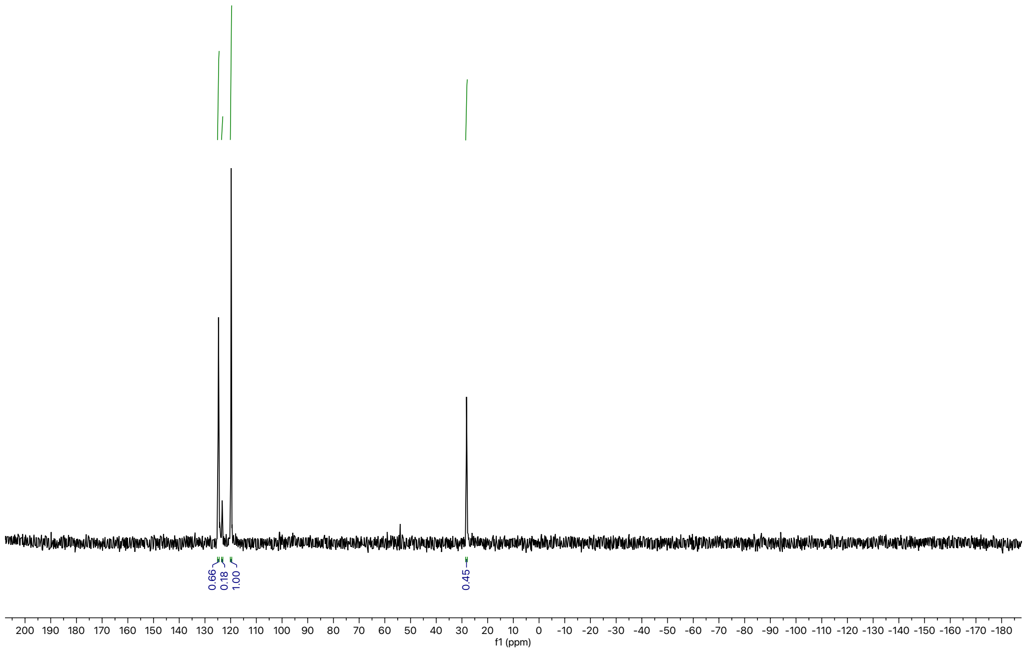

Figure S24. ^{31}P{^{1}H} NMR spectrum of the product obtained from the reaction between PN^{3}P-^{i}Pr and [Ru(PPh$_3$)$_3$HCl(CO)] in d_4-methanol at 298K.

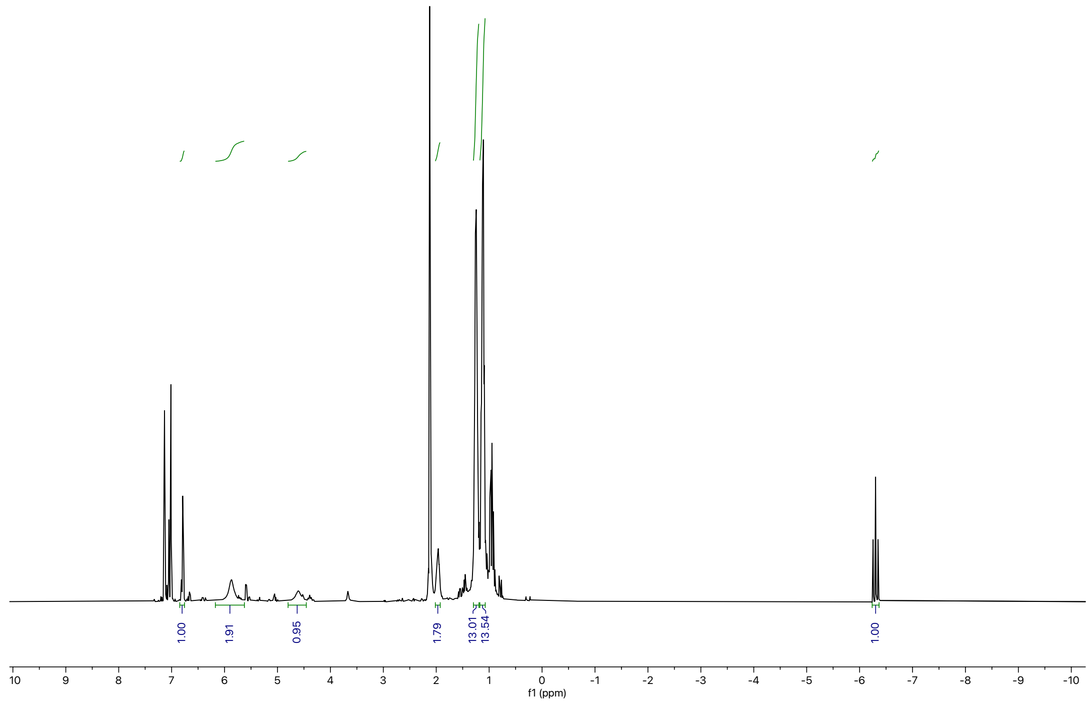

Figure S25. ^{1}H NMR spectrum of the product obtained from the reaction between PN^{3}P-^{i}Pr and $Ru_3(CO)_{12}$ in d_8-toluene at 298K.

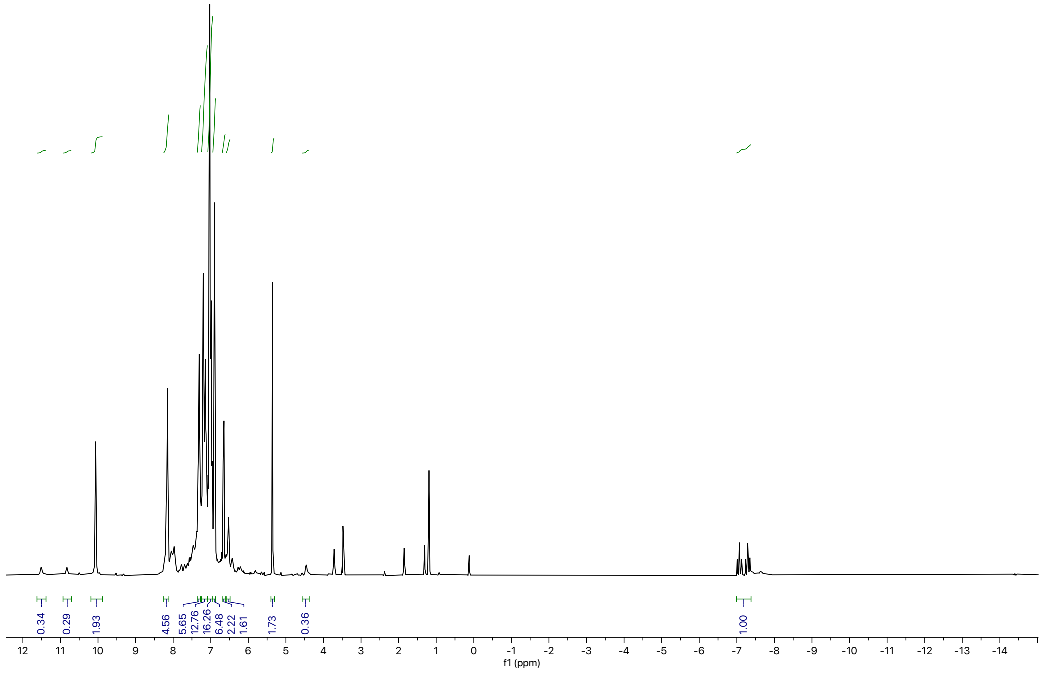

Figure S26. ^{1}H NMR spectrum of the product obtained from the reaction between PN^{3}P-Ph and [Ru(PPh_3)$_3$HCl(CO)] in CD_2Cl_2 at 298K. Assumed: [Ru(PN^{3}P-Ph)(PPh_3)HCl(CO)]Cl.

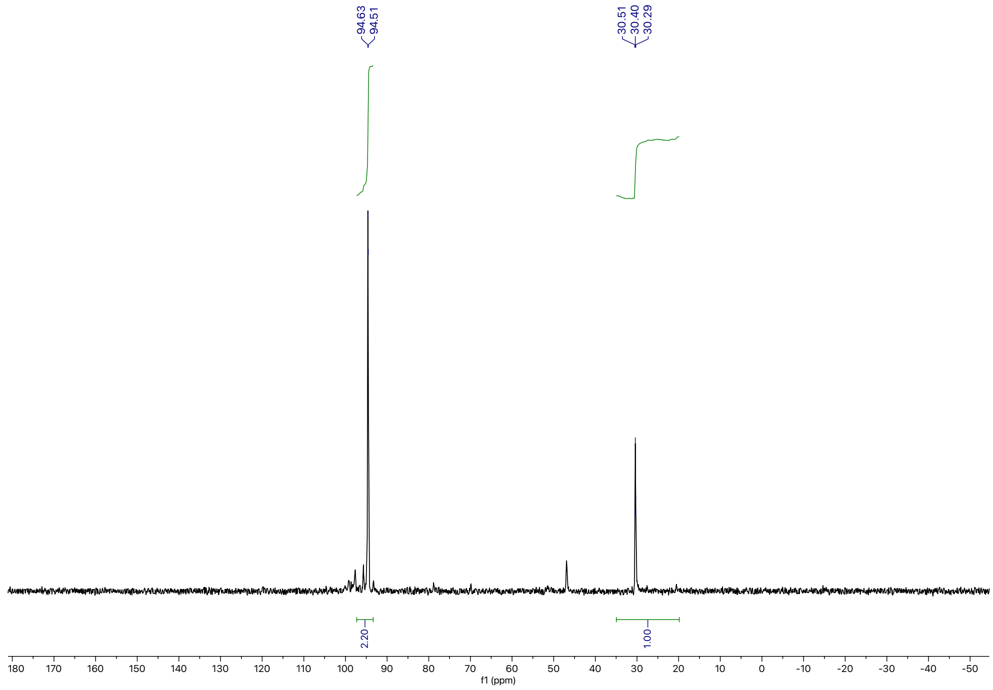

Figure S27. $^{31}P\{^{1}H\}$ NMR spectrum of the product obtained from the reaction between PN^{3}P-Ph and $[Ru(PPh_3)_3HCl(CO)]$ in CD_2Cl_2 at 298K.

IR Spectra

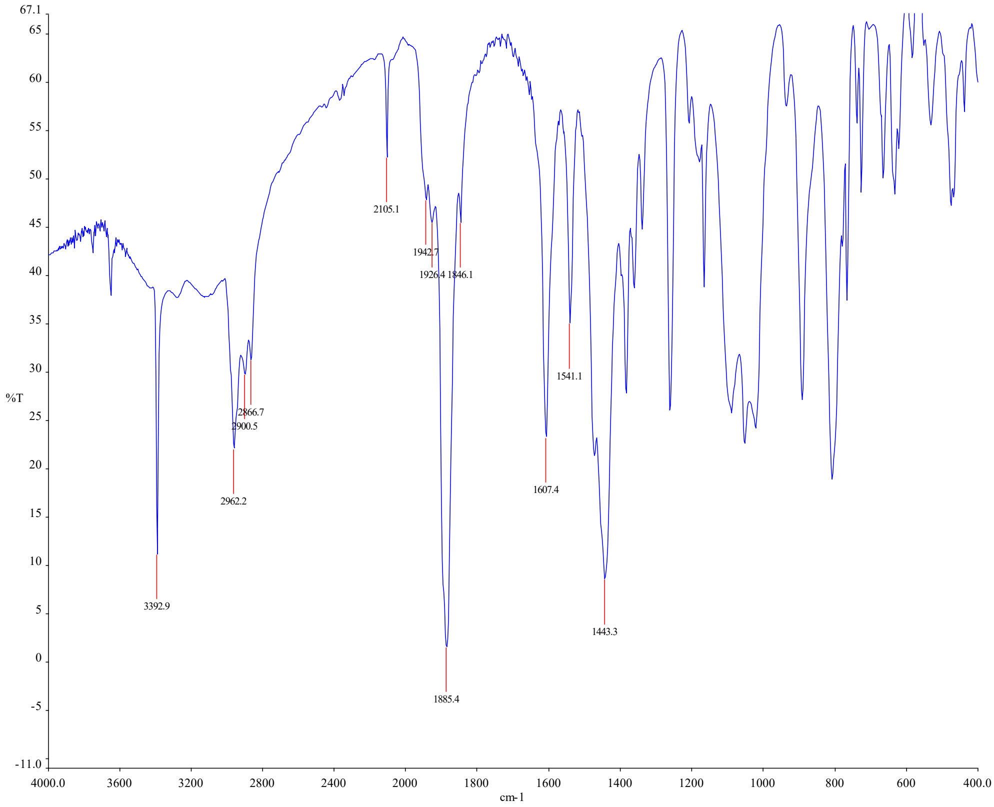

Figure S28. IR spectrum of complex [RuH(CO)(PN^{3}P-^{t}Bu-H)] (**3**) in KBr.

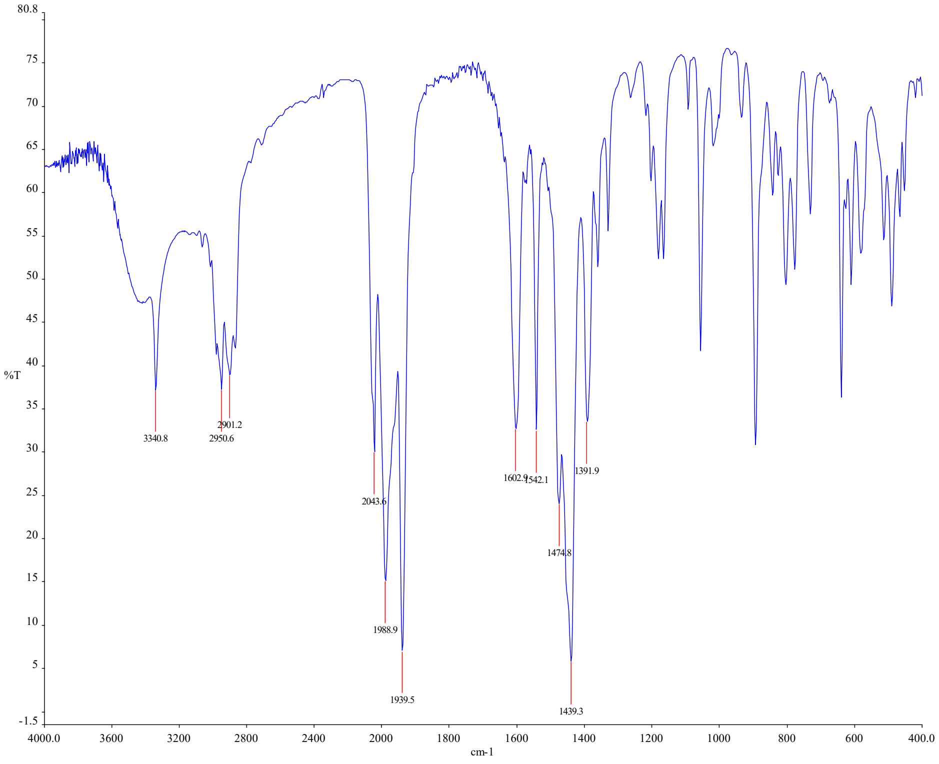

Figure S29. IR spectrum of complex [RuH(CO)$_2$(PN^{3}P-^{t}Bu-H)] (**5**) in KBr.

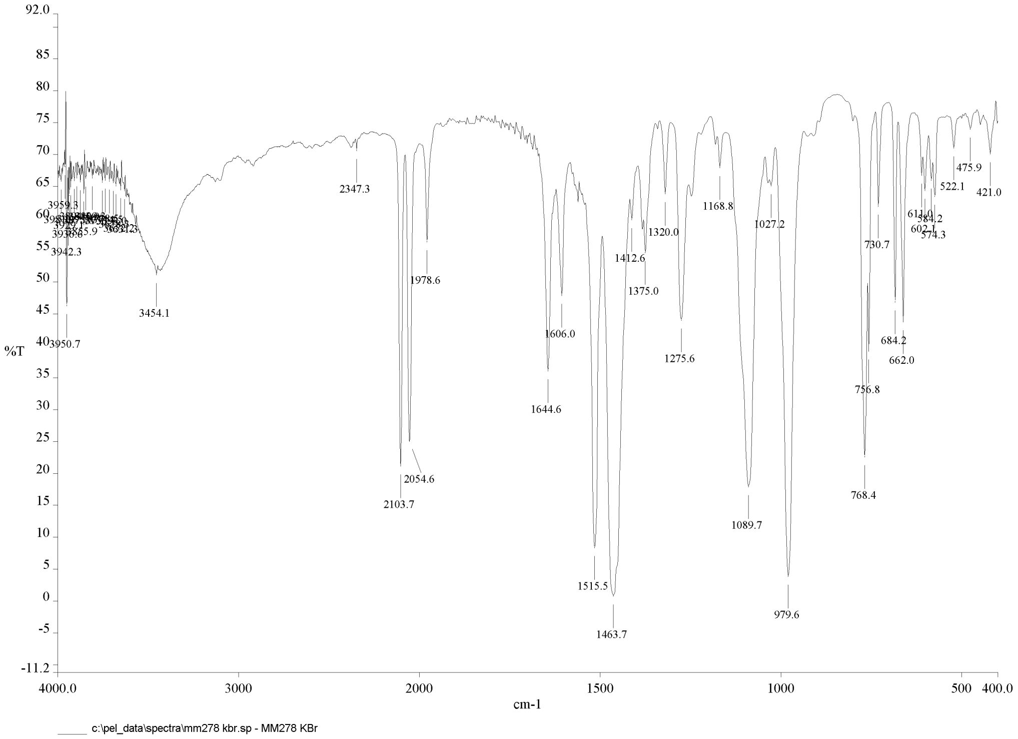

Figure S30. IR spectrum of complex [Ru(bipy)$_2$(CO)$_2$](B(C$_6$F$_5$)$_4$)$_2$ (**8**) in KBr.

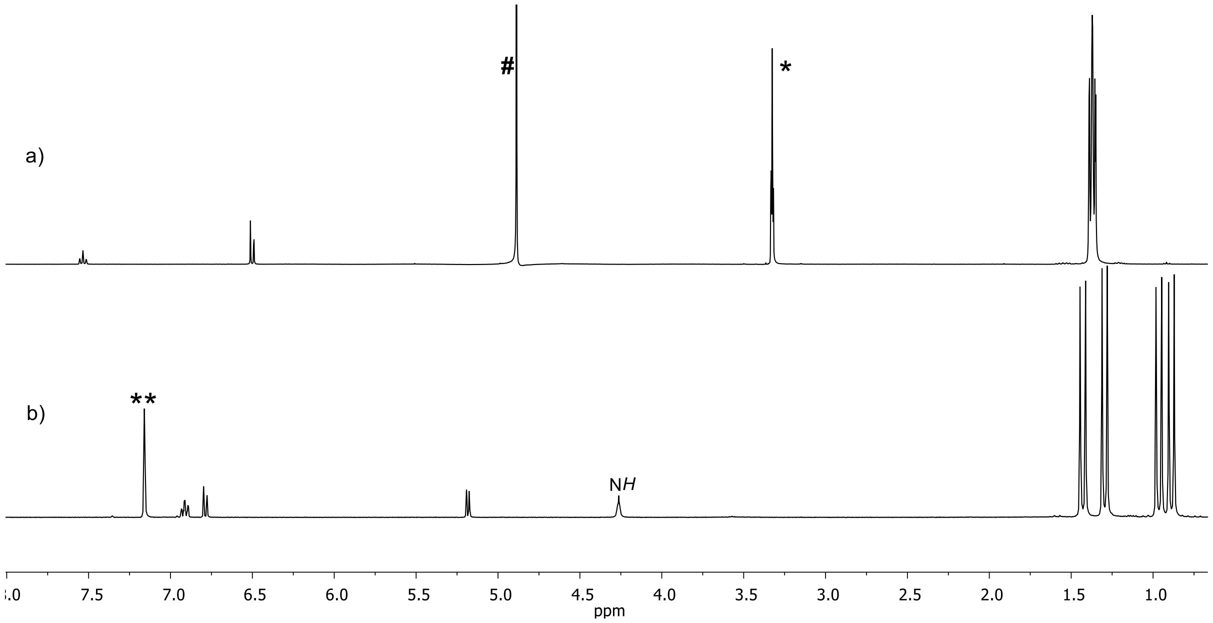

Figure S31: Comparison of ^{1}H NMR spectra at 298 K of a) complex [RuHCl(CO)(PN^{3}P-^{t}Bu)] (**1**) in d_4-MeOH (*) (# = H_2O) and b) complex [RuH(CO)(PN^{3}P-^{t}Bu-H)] (**3**) in d_6-benzene (**). Hydride signals omitted.

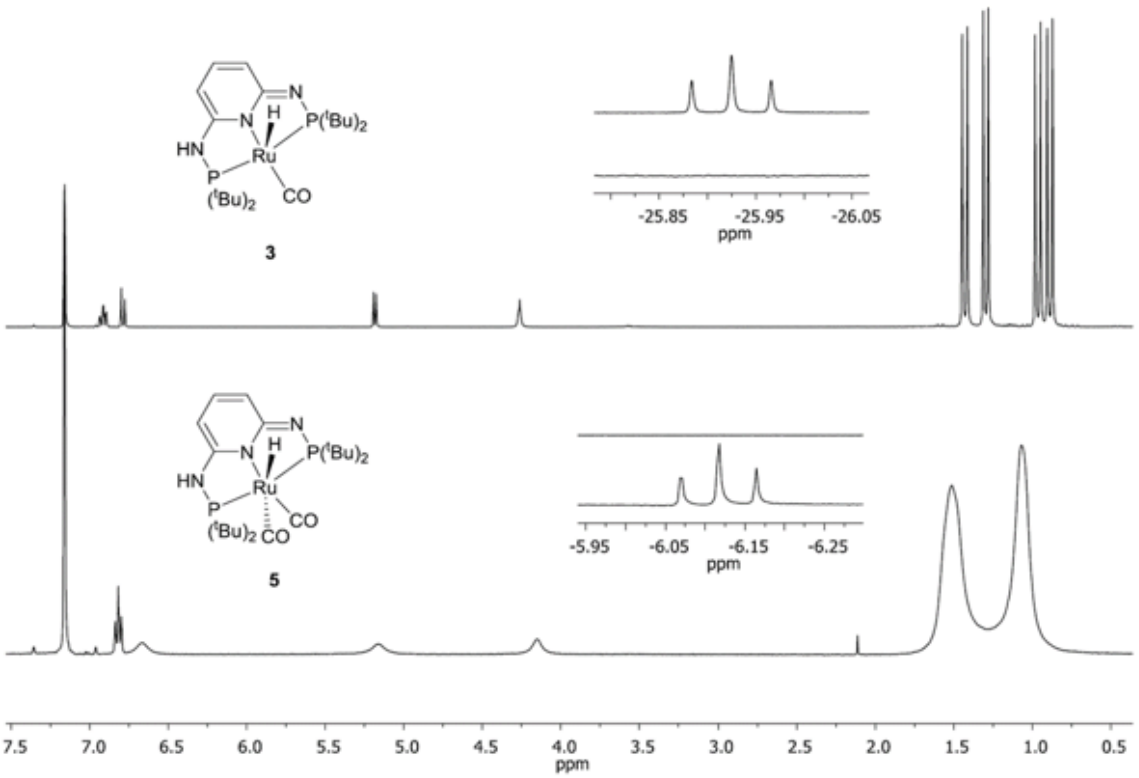

Figure S32: Comparison of the ^{1}H NMR spectra of [RuH(CO)(PN^{3}P-^{t}Bu-H)] (**3**) and [RuH(CO)$_2$(PN^{3}P-^{t}Bu-H)] (**5**) in d_6-benzene at 298 K.

The free energy of activation ($\Delta G^{\ddagger}$) for the exchange process in complex **5** was estimated from the NMR spectra in Figure 1, using Equation 1 below, wherein R is the gas constant (8.314 J mol^{-1}), Tc is the coalescence temperature, and $\Delta\nu$ is the difference in chemical shift between the exchanging environments.

$$\Delta G^{\ddagger} = RT_c\,[22.96 + ln\,(T_c/\Delta\nu)] \qquad (1)$$

The resolved *tert*-butyl signals are separated by 244 Hz ($\Delta\nu$) at 193K in the ^{1}H NMR spectrum, and coalesce at 373 K (T_c). This results in an estimated free energy of activation of 73 kJ mol^{-1}.

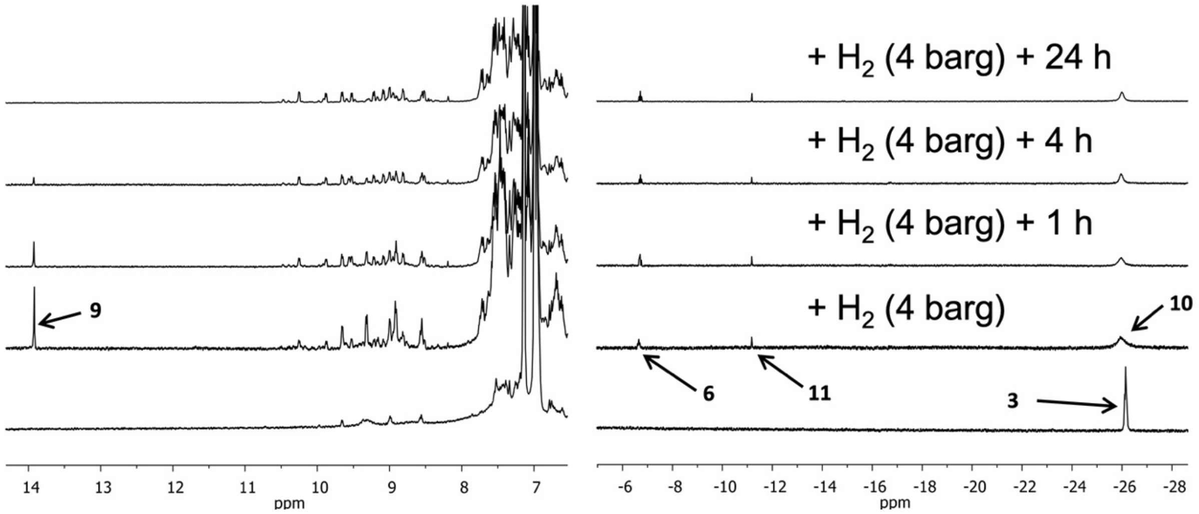

Figure S33. ^{1}H NMR spectra of the reaction between [Ru(bipy)$_2$(CO)$_2$](B(C$_6$F$_5$)$_4$)$_2$ (**8**) with [RuH(CO)(PN^{3}P-^{t}Bu-H)] (**3**), in the presence of H_2 (4 bar) in C$_6$D$_5$Cl at RT (as depicted in Scheme 7).

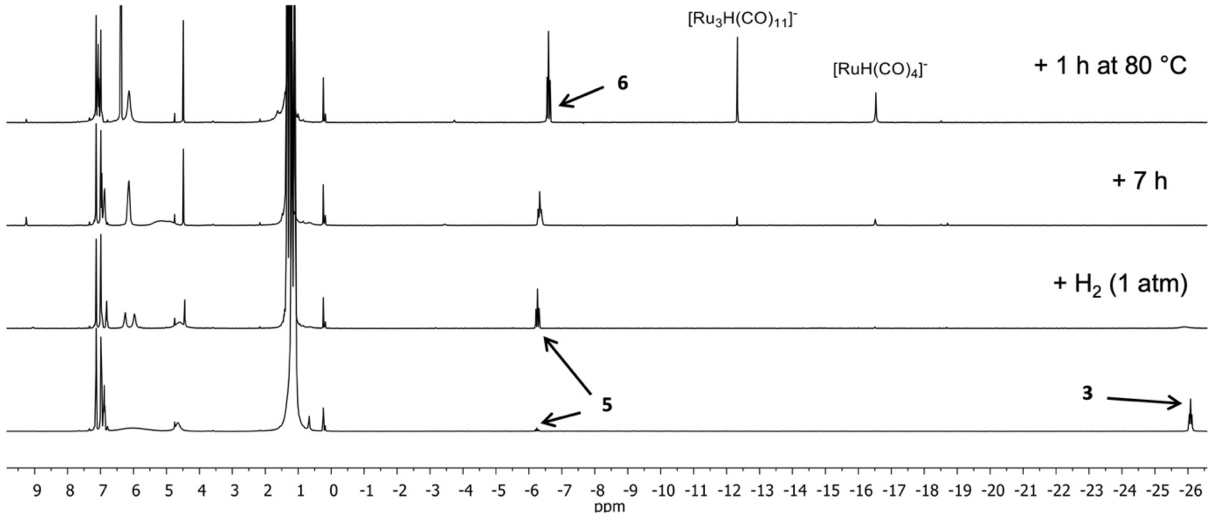

Figure S34. ^{1}H NMR spectra in C$_6$D$_5$Cl of the reaction of [RuH(CO)(PN^{3}P-^{t}Bu-H)] (**3**) with H_2 in the presence of a stoichiometric amount of [Ru$_3$(CO)$_{12}$] (as depicted in Scheme 8).

X-Ray Crystallography

The X-ray crystal structure of [RuH(CO)$_2$(PN^{3}P-^{t}Bu)]BArF

Crystal data for **[RuH(CO)$_2$(PN^{3}P-^{t}Bu)]BArF (6)**: [$C_{23}H_{42}N_3O_2P_2Ru$]($C_{32}H_{12}BF_{24}$)·C_6H_6, M = 1496.94, triclinic, P-1 (no. 2), a = 12.6966(5), b = 15.2947(6), c = 18.4280(7) Å, α = 96.400(3), β = 101.736(3), γ = 106.227(4)°, V = 3309.8(2) Å^3, Z = 2, D_c = 1.502 g cm^{-3}, μ(Cu-Kα) = 3.378 mm^{-1}, T = 173 K, colourless blocks, Agilent Xcalibur PX Ultra A diffractometer; 12608 independent measured reflections (R_{int} = 0.0346), F^2 refinement,[7, 8] R_1(obs) = 0.0663, wR_2(all) = 0.1722, 8880 independent observed absorption-corrected reflections [$|F_o| > 4\sigma(|F_o|)$, completeness to θ_{full}(67.7°) = 98.3%], 1006 parameters. CCDC 2260612.

Crystals of complex **[RuH(CO)$_2$(PN^{3}P-^{t}Bu)]BArF** (**6**) suitable for X-ray diffraction were obtained by slow diffusion of hexane into a benzene solution of the complex. The whole of the ruthenium complex in the structure of **[RuH(CO)$_2$(PN^{3}P-^{t}Bu)]BArF** was found to be disordered. Two orientations were identified of *ca*. 68 and 32% occupancy (approximately related by a mirror plane that would pass through N1, P8 and P10, see Fig. S36), their geometries were restrained to be similar, the thermal parameters of adjacent atoms were restrained to be similar, and only the non-hydrogen atoms of the major occupancy orientation, and the ruthenium atom of the minor occupancy orientation, were refined anisotropically (the remaining atoms were refined isotropically). The C45-, C53-, and C61-based CF_3 groups, and the C71-based included benzene solvent molecule, were all found to be disordered. In each case two orientations were identified, of *ca*. 78:22, 86:14, 77:23 and 77:23% occupancy respectively. The geometries of each pair of orientations were optimised, the thermal parameters of adjacent atoms were restrained to be similar, and only the non-hydrogen atoms of the major occupancy orientations were refined anisotropically (those of the minor occupancy orientations were refined isotropically). Unsurprisingly given the disorder of the whole of the ruthenium complex, the expected Ru–H hydride atom could not be located — its likely position *trans* to the C27-based carbonyl ligand would, for each orientation of the complex, be close to the ruthenium centre of the other orientation, so it would be utterly swamped by the electron density of the other Ru centre in the area.

Figures

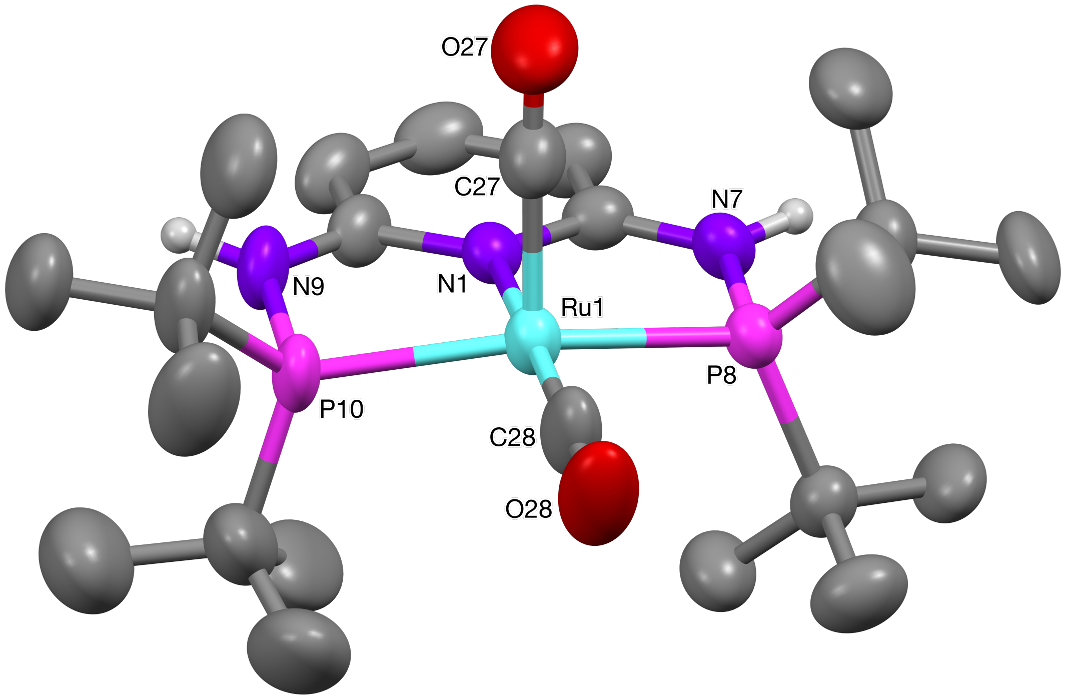

Fig. S35 The structure of the ruthenium complex present in the crystal of **[RuH(CO)$_2$(PN^{3}P-^{t}Bu)]BArF** (**6**) (50% probability ellipsoids).

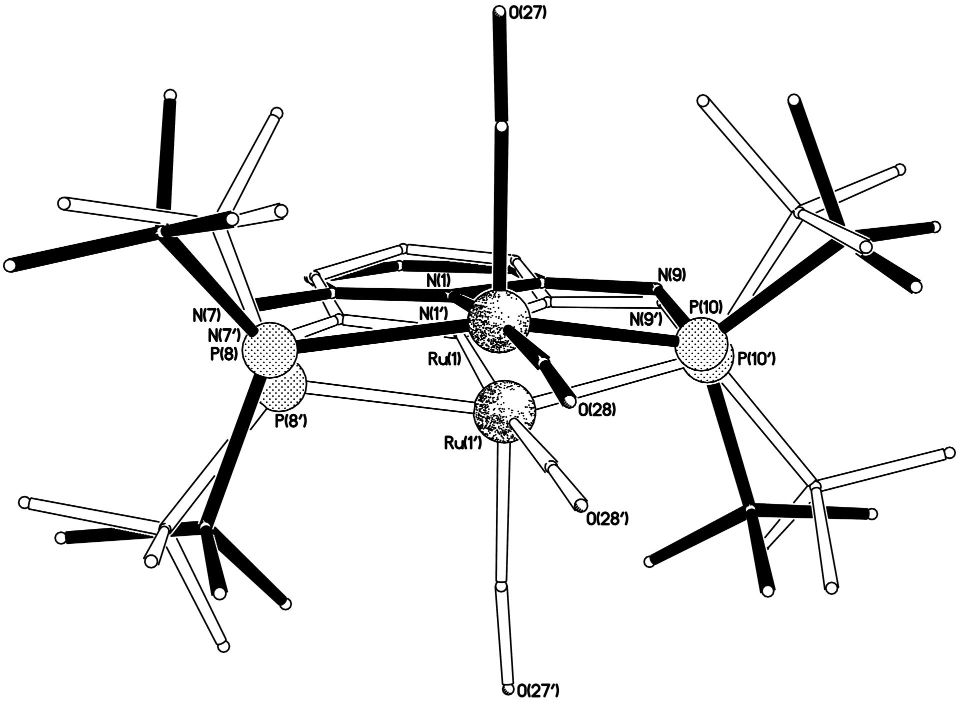

Fig. S36 The structure of the ruthenium complex present in the crystal of **[RuH(CO)$_2$(PN^{3}P-^{t}Bu)]BArF** showing the two partial occupancy orientations of the ruthenium complex (the major, *ca*. 68%, occupancy orientation has been drawn with dark bonds, whilst the minor, *ca*. 32%, occupancy orientation has been drawn with open bonds).

References

1. N. Ahmad, J. J. L., S. D. Robinson, M. F. Uttley, E. R. Wonchoba, G. W. Parshall, Complexes of Ruthenium, Osmium, Rhodium, and Iridium Containing Hydride Carbonyl, or Nitrosyl Ligands. *Inorg. Synth. 15*, 45-64.

2. Brookhart, M.; Grant, B.; Volpe, A. F., A convienient reagent for the generation and stabilisation of cationic, highly electrophilic organometallic complexes. *Organometallics* **1992,** *11* (11), 3920.

3. Benito-Garagorri, D.; Becker, E.; Wiedermann, J.; Lackner, W.; Pollak, M.; Mereiter, K.; Kisala, J.; Kirchner, K., Achiral and Chiral Transition Metal Complexes with Modularly Designed Tridentate PNP Pincer-Type Ligands Based on N-Heterocyclic Diamines. *Organometallics* **2006,** *25* (8), 1900-1913.

4. Schirmer, W.; Flörke, U.; Haupt, H. J., Zur Charakterisierung von Eigenschaften der versteiften dreizähnigen Aminophosphanliganden N,N'-Bis(diphenylphosphino)-2,6-diaminopyridin und N,N-Bis(diphenylphosphino)-2-aminopyridin mit Metallen der Chromgruppe. *Z. Anorg. Allg. Chem.* **1989,** *574* (1), 239-255.

5. Nobbs, J. D.; Sugiarto, S.; See, X. Y.; Cheong, C. B.; Aitipamula, S.; Stubbs, L. P.; van Meurs, M., Tetramethylphosphinane as a new secondary phosphine synthon. *Nature Commun.* **2023,** *6* (1), 85.
6. He, L.-P.; Chen, T.; Xue, D.-X.; Eddaoudi, M.; Huang, K.-W., Efficient transfer hydrogenation reaction Catalyzed by a dearomatized PN3P ruthenium pincer complex under base-free Conditions. *J. Organomet. Chem.* **2012,** *700*, 202-206.
7. Dolomanov, O. V.; Bourhis, L. J.; Gildea, R. J.; Howard, J. A. K.; Puschmann, H., OLEX2: a complete structure solution, refinement and analysis program. *J. Appl. Cryst.* **2009,** *42* (2), 339-341.
8. Sheldrick, G. M., SHELXT - integrated space-group and crystal-structure determination. *Acta Crystallog.r A Found Adv.* **2015,** *71* (Pt 1), 3-8.
